# Targeting necroptosis in MCF-7 breast cancer cells: *In Silico* insights into 8,12-dimethoxysanguinarine from *Eomecon Chionantha* through molecular docking, dynamics, DFT, and MEP studies

**DOI:** 10.1371/journal.pone.0313094

**Published:** 2025-01-07

**Authors:** Maram B. Alhawarri, Mohammad G. Al-Thiabat, Amit Dubey, Aisha Tufail, Katreen Banisalman, Ghazi A. Al Jabal, Eman Alkasasbeh, Esra’a Ibrahim Al-Trad, Bilal Harieth Alrimawi

**Affiliations:** 1 Faculty of Pharmacy, Department of Pharmacy, Jadara University, Irbid, Jordan; 2 Michael Sayegh Faculty of Pharmacy, Aqaba University of Technology, Aqaba, Jordan; 3 Department of Pharmacology, Saveetha Dental College and Hospital, Saveetha Institute of Medical and Technical Sciences, Chennai, Tamil Nadu, India; 4 Computational Chemistry and Drug Discovery Division, Quanta Calculus, Greater Noida, Uttar Pradesh, India; 5 Faculty of Pharmacy and Biomedical Sciences, Department of Medicinal Chemistry, MAHSA University, Jenjarom, Selangor, Malaysia; 6 Faculty of Applied Medical Sciences, Department of Medical Laboratory Sciences, Al al-bayt University, Mafraq, Jordan; Al-Azhar University Faculty of Pharmacy for Boys, EGYPT

## Abstract

Breast cancer remains a significant challenge in oncology, highlighting the need for alternative therapeutic strategies that target necroptosis to overcome resistance to conventional therapies. Recent investigations into natural compounds have identified 8,12-dimethoxysanguinarine (SG-A) from *Eomecon chionantha* as a potential necroptosis inducer. This study presents the first computational exploration of SG-A interactions with key necroptotic proteins—RIPK1, RIPK3, and MLKL—through molecular docking, molecular dynamics (MD), density functional theory (DFT), and molecular electrostatic potential (MEP) analyses. Molecular docking revealed that SG-A exhibited a stronger affinity for MLKL (-9.40 kcal/mol) compared to the co-crystallized ligand (-6.29 kcal/mol), while its affinity for RIPK1 (-6.37 kcal/mol) and RIPK3 (-7.01 kcal/mol) was lower. MD simulations further demonstrated the stability of SG-A within the MLKL site, with RMSD values stabilizing between 1.4 and 3.3 Å over 300 ns, indicating a consistent interaction pattern. RMSF analysis indicated the preservation of protein backbone flexibility, with average fluctuations under 1.7 Å. The radius of gyration (Rg) results indicated a consistent value of ~15.3 Å across systems, confirming the role of SG-A in maintaining protein integrity. Notably, SG-A maintains two critical H-bonds within the active site of MLKL, reinforcing the stability of the interaction. Principal component analysis (PCA) indicated a significant reduction in MLKL’s conformational space upon SG-A binding, implying enhanced stabilization. Dynamic cross-correlation map (DCCM) analysis further revealed that SG-A induced highly correlated motions, reducing internal fluctuations within MLKL compared to the co-crystallized ligand. MM-PBSA revealed the enhanced binding efficacy of SG-A, with a significant binding free energy of -31.03 ± 0.16 kcal/mol against MLKL, surpassing that of the control (23.96 ± 0.11 kcal/mol). In addition, the individual residue contribution analysis highlighted key interactions, with ARG149 showing a significant contribution (-176.24 kcal/mol) in the MLKL-SG-A complex. DFT and MEP studies corroborated these findings, revealing that the electronic structure of SG-A is conducive to stable binding interactions, characterized by a narrow band gap (~0.16 units) and distinct electrostatic potential favourable for necroptosis induction. In conclusion, SG-A has emerged as a compelling inducer of necroptosis for breast cancer therapy, warranting further experimental validation to fully realize its therapeutic potential.

## 1. Introduction

Breast cancer remains one of the most prevalent and devastating forms of cancer worldwide [1]. According to the World Health Organization, breast cancer is responsible for one in every eight cancer diagnoses, with an estimated 2.3 million new cases in 2020, highlighting its widespread impact and the ongoing need for effective treatments [2,3]. The therapeutic approaches for breast cancer encompass a diverse array of modalities, including surgical intervention, radiotherapy, chemotherapeutic regimens, hormonal treatments, and precision-targeted therapies [4]. However, the efficacy of present therapeutic approaches is frequently compromised by the intrinsic heterogeneity of breast cancer and the development of resistance to current treatments [5]. This phenomenon is particularly relevant for estrogen receptor-positive (ER+) breast cancers, such as those associated with the MCF-7 cell line, which are prone to resistance against these therapies and require specialized hormonal treatments [6]. Therefore, there is an urgent need for the development of new therapeutic agents that are effective and safe, especially for ER+ breast cancers, which are highly likely to be resistant [7]. While apoptosis serves as a critical defence mechanism against cancer by promoting programmed cell death, the ability of cancer cells to evade or become resistant to this process is a well-documented hallmark of cancer progression and treatment resistance [8]. This resistance to apoptosis is a significant obstacle in oncology, often leading to the failure of chemotherapeutic interventions [9]. Consequently, interest in the exploration of alternative pathways of programmed cell death, such as necroptosis [10], which could offer new avenues for cancer therapy, is increasing.

Historically, necrosis was perceived as an unregulated and chaotic form of cell death, distinct from the orderly process of apoptosis [11]. This perspective shifted dramatically following groundbreaking work by Vercammen et al. in 1998 [12] and further insights from Degterev et al. in 2005 [13], who coined the term ’necroptosis’ for a form of regulated necrosis. Necroptosis is characterized by specific morphological changes, such as cellular swelling, membrane rupture, and the eventual release of cellular contents, distinguishing it clearly from the apoptotic pathway, which is known for nuclear condensation and membrane integrity [14,15]. Biochemically, necroptosis involves no active protein-related processes comparable to the hallmarks of apoptosis, such as caspase activation or the caspase-mediated cleavage of cellular proteins [15,16]. The molecular mechanism underlying necroptotic cell death is characterized by the activation and integration of a trio of downstream signalling pathways formed by receptor-interacting protein kinase 1 (RIPK1), receptor-interacting protein kinase 3 (RIPK3), and mixed lineage kinase domain-like protein (MLKL) [15,17]. These proteins collaboratively facilitate a cascade that initiates and regulates cell death and potentially plays a critical role in the necroptosis process [15, 17].

The investigation of necroptosis as a therapeutic target has led to the discovery of various drug candidates and herbal treatments that specifically modulate RIPK1, RIPK3, and MLKL [18]. Among these, FDA-approved inhibitors such as necrostatin-1 (Nec-1) have shown significant promise in targeting RIPK1 [19,20], suggesting potential therapeutic options for diseases characterized by excessive necroptosis [19,20]. GSK’872, a potent RIPK3 inhibitor, has been extensively studied in preclinical models, and its efficacy in suppressing necroptosis has been demonstrated [21,22]. Furthermore, MLKL, the terminal effector in the necroptotic pathway, has emerged as a key target, with compounds such as necrosulfonamide (NSA) exhibiting selective inhibition of MLKL oligomerization [23,24]. Additionally, natural compounds such as curcumin and resveratrol have been reported to modulate necroptotic pathways [25–27], highlighting the therapeutic potential of herbal treatments in regulating necroptosis. These existing therapeutic strategies underscore the critical role of RIPK1, RIPK3, and MLKL in the development of new anticancer agents, particularly for malignancies that are resistant to apoptosis [28,29].

**Fig 1:**
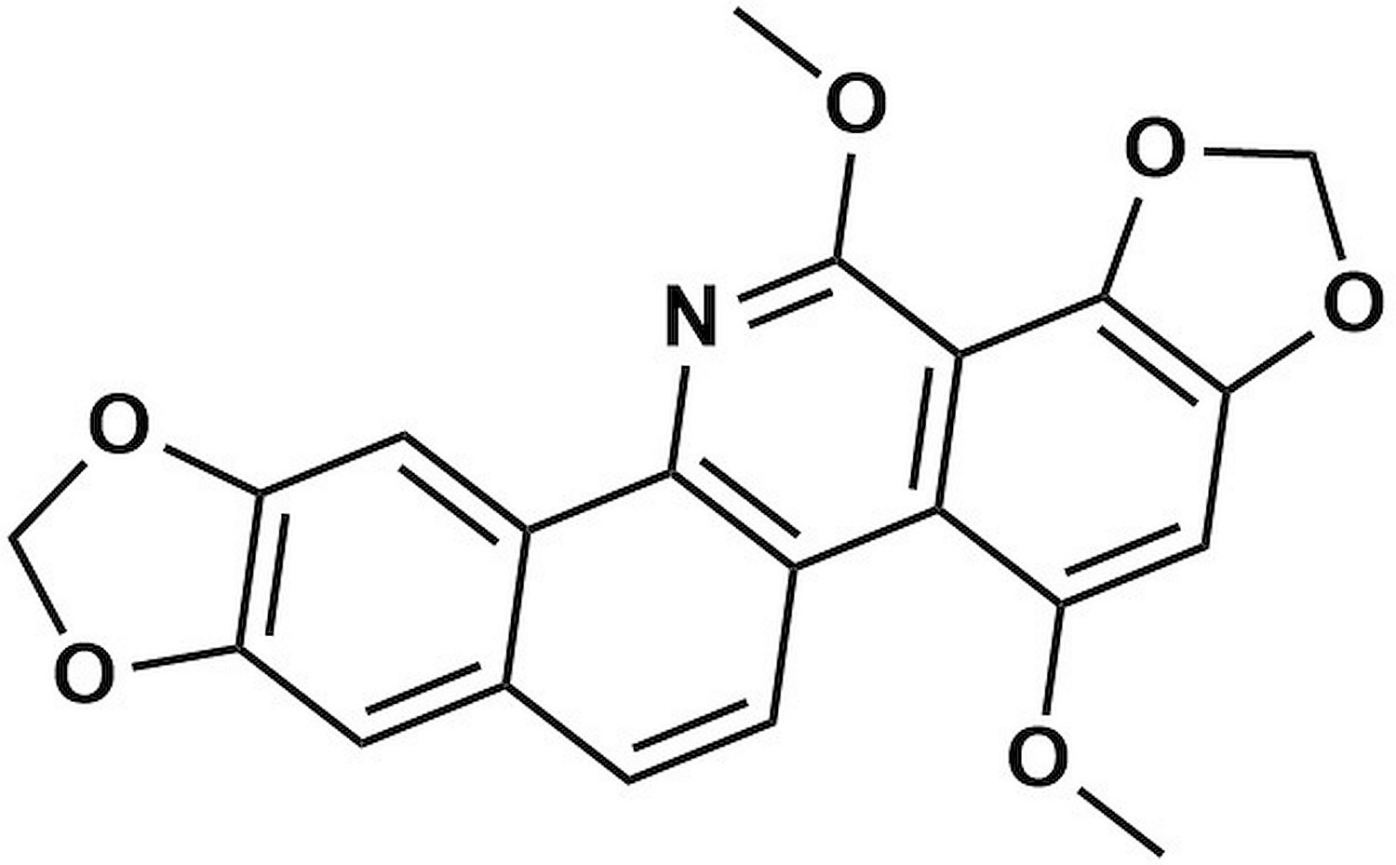


**Fig 2:**
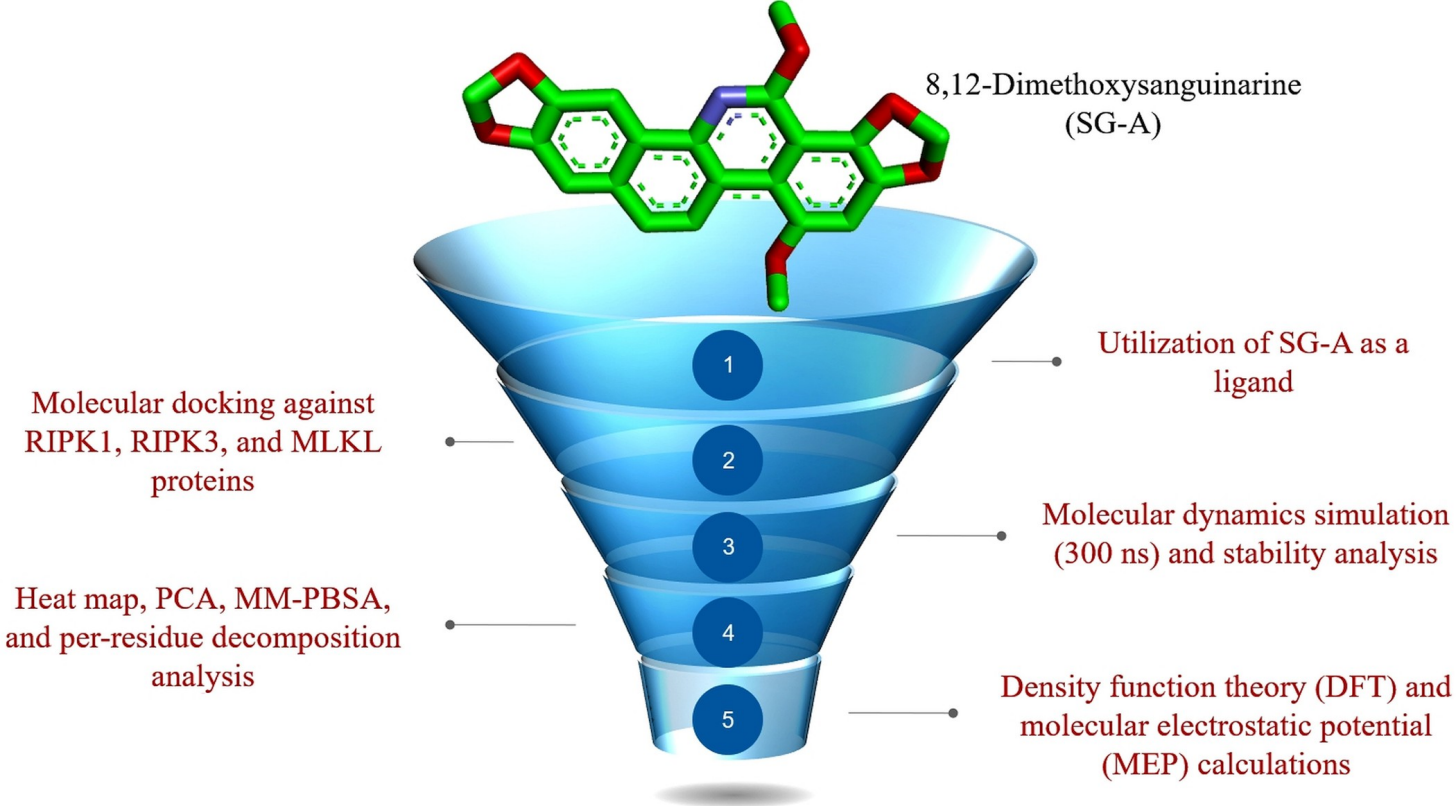


Despite advancements in breast cancer therapy, the search for anticancer compounds with enhanced effectiveness, safety, and selectivity continues to be crucial [30]. Within this context, the exploration of natural products as sources of anticancer agents represents a relatively unexplored avenue for novel drug development owing to their extensive chemical diversity and multifactorial biological mechanisms[31–33]. Recently, a new benzophenanthridine alkaloid, 8,12-dimethoxysanguinarine (SG-A) (Fig 1), was isolated from *Eomecon chionantha* (Papaveraceae) [34]. When tested against the MCF-7 breast cancer cell line, SG-A exhibited a notable ability to initiate nonapoptotic cell death through necroptosis [35]. This phenomenon was demonstrated through flow cytometry, Hoechst 33258 staining, and transmission electron microscopy (TEM) analyses, which provided insights into changes in cell morphology [22]. These observations highlight the potential of SG-A to trigger necroptotic cell death, revealing an innovative therapeutic path that diverges from the conventional focus on apoptosis in cancer treatment [22]. However, the specific impacts of SG-A on the principal pathways involved in necroptotic cell death, namely, RIPK1, RIPK3, and MLKL, remain unexplored through experimental or computational studies. To address this gap, this study is the first to explore the potential affinity and molecular interaction stability of SG-A against these proteins *in silico* approaches such as molecular docking, molecular dynamics (MD), density functional theory (DFT), and molecular electrostatic potential (MEP) methods. A summary of the workflow used to explore the role of SG-A in the necroptosis pathway is shown in Fig 2. These approaches may enable researchers to identify and design effective drug compounds more rapidly and efficiently than traditional experimental methods do by predicting the binding affinity, molecular interactions, stability, electronic structure and reactivity of a compound to specific receptors or enzymes [36,37]. Consequently, the main objective of this research was to computationally investigate the ability of the newly identified benzophenanthridine alkaloid SG-A from *Eomecon chionantha* to initiate necroptosis. This study sought to elucidate the therapeutic potential of SG-A in activating necroptosis, with the goal of establishing a foundational understanding of necroptotic cell death that not only enhances our current research but also significantly aids in the development of targeted and more efficacious therapeutic strategies.

## 2. Materials and methods

### 2.1 Molecular docking simulation

The molecular docking simulation conducted in this study aimed to elucidate the potential interactions between SG-A and key proteins involved in the necroptosis pathway, specifically RIPK1, RIPK3, and MLKL [15,38]. The X-ray crystallographic structures of the protein targets RIPK1 (PDB ID: 6NYH) [39], RIPK3 (PDB ID: 7MON) [40], and MLKL (PDB ID: 6ZZ1) [41], along with their associated co-crystallized ligands, were retrieved from the RCSB Protein Data Bank (https://www.rcsb.org) for detailed binding interaction studies [42]. These structures were downloaded on March 5^th^, 2024, for RIPK1 and MLKL and on August 5^th^, 2024, for RIPK3. To maintain the precision of the structural analysis, all water molecules and heteroatoms were eliminated via the Biovia Discovery Studio Visualizer (San Diego, CA, USA, 2019) [43], a crucial step for minimizing inaccuracies [44,45]. The proteins were then subjected to molecular docking through the PDB2PQR web service (https://pdb2pqr.poissonboltzmann.org/pdb2pqr) [46], which was accessed on March 7^th^, 2024, for RIPK1 and MLKL and on August 6^th^, 2024, for RIPK3. This service facilitated the reconstruction of missing atoms and the assignment of atomic charges and radii according to the SWANSON force field (employing AMBER ff99 charges with optimized radii) [44,45,47–51]. The protonation states of the ionizable groups were established via the empirical pKa predictor PROPKA3, which was set at pH 7.4, to simulate physiological conditions [45,50,52,53]. Subsequent to protonation, the proteins underwent a refinement process via the MolProbity web service (http://molprobity.biochem.duke.edu/) on March 7^th^, 2024, for RIPK1 and MLKL, and on August 6^th^, 2024, for RIPK3, to correct atomic contacts and append missing hydrogen atoms [54], ensuring structural analysis integrity and accuracy [44,45].

In this study, the newly identified benzophenanthridine alkaloid SG-A (ligand) was sketched via PerkinElmer ChemDraw Professional 17.1 software (PerkinElmer, Massachusetts, USA) [45,55–60]. Energy minimization of the ligand was carried out via the molecular mechanics 2 (MM2) force field implemented in PerkinElmer Chem3D 17.1 software (PerkinElmer, Waltham, MA, USA) [45,55–60]. The minimized structure of the ligand was then saved in PDB format. SG-A was then utilized as a ligand in the molecular docking process. The co-crystallized ligands of the selected protein targets were extracted from the complexes and saved as PDB files via the Biovia Discovery Studio Visualizer (San Diego, CA, USA, 2019) [45,55–60]. Gasteiger charges were assigned to the ligand via AutoDockTools 1.5.6 (The Scripps Research Institute, San Diego, CA, USA) [61,62]. The ligand was subsequently redocked to the protein as a control via AutoDock 4.2 for comparison with the obtained ligands [45,55–60].

AutoDockTools 1.5.6 facilitated the preparation of proteins for the docking simulations [61]. The proteins underwent a modification to include polar hydrogen charges [45,55–60]. These charged entities were then saved in the PDBQT file format for further processing [45,55–60]. Active binding sites are specific regions of macromolecules where a ligand molecule can bind and potentially exert the desired biological activity [63]. In the present study, grid box coordinates were established on the basis of co-crystallized ligand coordinates for the analysis of protein‒ligand interactions, and the coordinates are listed in S1 Table.

**Fig 3:**
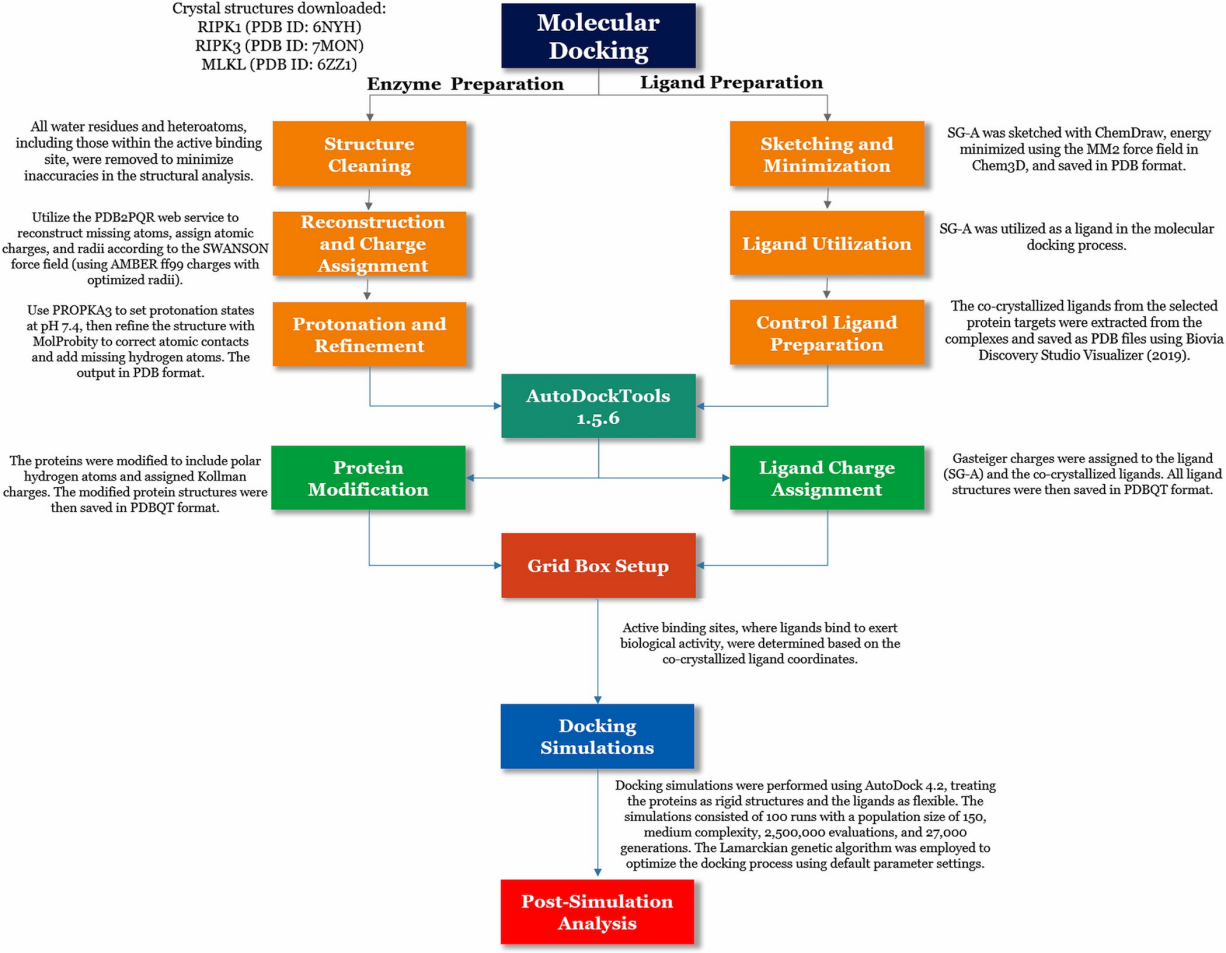


AutoDock 4.2 was used to carry out the docking simulations [61], treating proteins as rigid structures and ligands as flexible entities. This simulation encompassed 100 docking iterations, employing a population size of 150, a medium complexity level with 2,500,000 evaluations, and a maximum of 27,000 generations [45,55,56,64]. The Lamarckian genetic algorithm was chosen for these simulations [62,65], with all other parameters remaining at their default settings, encapsulated within the docking parameter files (DPFs). For the post-simulation analysis, the Biovia Discovery Studio Visualizer (San Diego, CA, USA, 2019) was used to scrutinize and depict the molecular interactions between ligands and proteins through both two-dimensional and three-dimensional visualizations [43]. A summary workflow for the molecular docking simulation is presented in Fig 3.

### 2.2 Molecular dynamics simulation

In this study, our goal was to evaluate the stability and binding dynamics of SG-A within the active site of the MLKL protein over a 300 ns molecular dynamics (MD) simulation period. We initiated our simulations using the crystal structure of the MLKL protein complexed with its co-crystallized ligand as a reference point. The study also included simulations of the MLKL protein in its apo form (free ligand) and when bound to the co-crystallized ligand for the specified interval. For these MD simulations, we utilized the GROMACS software package (version 2016.3) alongside the Gromos96 54a7 force field [66]. The choice of the Gromos96 54a7 force field is grounded in its demonstrated reliability for analysing protein dynamics and ligand interaction behaviours, as established in previous studies [67]. The efficacy of this force field was further underscored by its successful application in simulating the folding equilibrium of two β-peptides, which exhibit distinct dominant folds across three different force fields, including GROMOS 54A7 [67].

For simulation preparation, topology files for both the protein and ligand were generated using specific computational tools. The GROMACS utility pdb2gmx was employed to construct the protein topology file, while the ligand topology was developed through the PRODRG server (http://davapc1.bioch.dundee.ac.uk/cgi-bin/prodrg, accessed on March 10^th^, 2024). Following this, the systems underwent solvation via the TIP3P water model, accompanied by the addition of counterions to achieve charge neutrality. Optimization of the systems was carried out using the steepest descent method, with up to 50,000 steps of optimization and an energy step size of 0.01. Temperature equilibration was achieved via the v-rescale method, maintaining a constant number of particles, volume, and temperature (NVT ensemble) for 100 ps at 310 K. This was succeeded by a 100 ps pressure equilibration phase under the Berendsen pressure coupling method at a constant 1.0 bar, adhering to a steady number of particles, pressure, and temperature (NPT ensemble) [68,69].

Following the temperature and pressure stabilization phases, MD simulation runs for the models were executed over 300 ns each, maintaining conditions at 1 bar and 310 K. The threshold for short-range nonbonded interactions was established at 1.2 nm, and long-range electrostatic interactions were addressed via the particle mesh Ewald (PME) method [70]. Bond constraints involving hydrogen atoms were managed with the LINCS algorithm [71]. A consistent time step of 2 fs was applied across all the simulations to ensure the precision of the MD simulations. The simulation data were meticulously managed by resetting the coordinates every 5000 steps, corresponding to 10 ps, to maintain the integrity of the simulation and ensure reliable outcomes. To dissect the MD trajectories, a suite of analytical tools and methodologies, including root mean square deviation (RMSD), root mean square fluctuation (RMSF), radius of gyration (Rg), and hydrogen bond (H-bond) analysis, were employed. These techniques facilitate a comprehensive evaluation of the interaction dynamics and stability between SG-A and the target protein, aiming to elucidate the efficacy of these interactions. The entire simulation was executed on a Linux operating system: Ubuntu 22.04.2 LTS 64-bit, Intel Xeon W-2245 @ 3.90 GHz, 12-Cores, CUDA 12, and NVIDIA RTX 4090.

### 2.3 MM-PBSA calculations

In our research, the molecular mechanics Poisson‒Boltzmann surface area (MM-PBSA) technique [72] was employed to determine the binding free energies from MD trajectory snapshots. This method, recognized for its ability to calculate binding energies, facilitated the analysis of binding free energies between SG-A and the control (co-crystallized ligand) in relation to the MLKL protein. The evaluations were conducted during the MD simulation production phase, with snapshots being taken every 100 ps from 270 to 300 ns. This analysis was performed utilizing the g_mmpbsa tool within the GROMACS suite [73,74], providing insights into the interaction strength of SG-A compared with that of the control ligand with the MLKL protein. The equations for calculating the binding free energy of the ligand‒protein complex in a solvent, which is essential for the MM-PBSA method, have been extensively described [57,75,76].

### 2.4 Density function theory (DFT) and molecular electrostatic potential (MEP) calculations

Density functional theory (DFT) has emerged as a dependable and cost-efficient technique for revealing fundamental insights into material properties, ranging from energy profiles and geometric structures to electrical attributes and optical characteristics [48]. This versatile approach is essential for interpreting data across various scales, spanning from the microscopic realm of atoms and molecules to larger unit cells [77]. In this study, DFT played a crucial role in thoroughly exploring parameters pivotal to electronic behavior, energetics, thermodynamics, and adsorption phenomena, with a specific emphasis on binding energy [78,79]. Additionally, investigations aimed to elucidate the reactivity of pharmacological complexes by employing quantum molecular descriptors, such as the highest occupied molecular orbital (HOMO), lowest unoccupied molecular orbital (LUMO), bandgap energy, chemical hardness, softness, electronegativity, and electrophilicity [48,80–82]. The Gaussian 09 software package was utilized as a computational tool to optimize the geometry of the molecular structures associated with SG-A, employing density functional three-parameter hybrid (B3LYP) methods along with the 6-311G (d, p) basis set [48]. This approach ensured the derivation of precise and meaningful insights into the molecular characteristics, providing a profound understanding of the benzophenanthridine alkaloid derivatives and their interactions within the pharmacological context. Furthermore, a molecular electrostatic potential (MEP) diagram for the salt was generated via the B3LYP-D3/6-311G (d, p) method [48]. This diagram serves as a visual representation of the electrostatic potential (ESP) superimposed onto the electron density (ED) surface, exhibiting a gradient of colours from the deepest red to the deepest blue [48]. This graphical representation effectively illustrates the electrostatic characteristics of the salt molecule, offering valuable insights into the distribution of electric charge across its surface.

## 3. Results and discussion

### 3.1 Molecular docking studies

Molecular docking stands as a cornerstone in the field of computational drug discovery, offering profound insights into the binding interactions between ligands and their protein targets [83]. This technique enables the prediction of ligand orientation within a protein’s binding site, elucidating key interactions and binding affinities that underpin molecular mechanisms of action [84]. Such computational analyses are indispensable for understanding the therapeutic potential of novel compounds, guiding the rational design of drugs with enhanced efficacy and specificity [84].

Initially, the molecular docking protocol was validated by redocking the original co-crystallized ligands within the active binding sites of the RIPK1, RIPK3, and MLKL protein structures, with PDB IDs of 6NYH (S1 Fig), 7MON (S2 Fig), and 6ZZ1 (S3 Fig), respectively. Table 1 presents the results of the free binding energy from the docking simulations performed via AutoDock 4.2 for SG-A and the co-crystallized ligands (used as controls) within the active sites of these three proteins. The analysis demonstrated that our methodology could accurately reproduce the conformation of the controls, as observed in their crystal structures, achieving root-mean-square deviation (RMSD) values of approximately 0.67 Å for RIPK1, 1.22 Å for RIPK3, and 1.13 Å for MLKL. RMSD values less than 2 Å are considered acceptable and indicate that AutoDock 4.2 successfully repositioned the co-crystallized structures while preserving the key interactions [49–51,56,85,86]. This validation supports the applicability of the docking protocol for studying the interactions between SG-A and its target proteins.

**Table 1:** Free binding energy (kcal/mol) of the new benzophenanthridine alkaloid (8,12-dimethoxysanguinarine; SG-A) and the co-crystallized ligands against the key proteins involved in the necroptosis pathway, particularly RIPK1, RIPK3, and MLKL.

Compounds	Key proteins involved in the necroptosis pathway		
	RIPK1 (*6NYH)	RIPK3 (*7MON)	MLKL (*6ZZ1)
**SG-A**	-6.37	-7.01	-9.40
* **Co-crystalized ligand**	-13.23	-9.84	-6.29

*6NYH: Structure of human RIPK1 kinase domain in complex with GNE684. *6ZZ1: Human crystal structure of MLKL executioner domain in complex with the co-crystalized ligand (7-(2-methoxyethoxymethyl)-1,3-dimethyl-purine-2,6-dione)). *7MON: Structure of human RIPK3 in complex with N-[4-(2-[(cyclopropanecarbonyl)amino]pyridin-4-yl)oxy)-3-fluorophenyl]-1-(4-fluorophenyl)-2-oxo-1,2-dihydropyridine-3-carboxamide.

The molecular docking results, detailed in Table 1, provide a comparative analysis of the binding affinities (free binding energies) between 8,12-dimethoxysanguinarine (SG-A) and the respective co-crystallized ligands for RIPK1, RIPK3, and MLKL. These results shed light on the interactions of SG-A with key proteins involved in the necroptosis pathway. For RIPK1 (PDB ID 6NYH), SG-A displayed a free binding energy of -6.37 kcal/mol, which is notably higher (less negative) than the free binding energy of the co-crystallized ligand GNE684 (-13.23 kcal/mol). This significant difference suggests that SG-A exhibits a weaker affinity for RIPK1, likely due to its rigid structure characterized by only two rotatable bonds. This rigidity may hinder SG-A’s ability to adapt conformationally to RIPK1’s “L”-shaped and narrow binding site [87], in contrast to the co-crystallized ligand GNE684, which likely has greater flexibility and can better accommodate the binding pocket. As shown in S4 Fig, SG-A does not engage in key interactions with essential residues within the active site, such as LYS45, MET92, VAL76, ASP156, LEU157, and PHE162 [88–90], which are critical for the co-crystallized ligand’s binding (S1 Fig). This lack of interaction with key residues further underscores SG-A’s lower binding affinity for RIPK1.

For RIPK3 (PDB ID 7MON), SG-A showed a free binding energy of -7.01 kcal/mol, which is also higher (less negative) than the co-crystallized ligand (-9.84 kcal/mol). Although SG-A is capable of binding to RIPK3, it does not fully accommodate the active site as effectively as the co-crystallized ligand. The binding pose analysis, as demonstrated in S5 Fig, reveals that SG-A is unable to form critical interactions with key residues such as SER101, PHE96, and LEU149 [91,92], which are crucial for stabilizing the co-crystallized ligand within the binding pocket (S2 Fig). The rigidity of SG-A may again impede its ability to adopt a conformation that optimally interacts with the active site, explaining its relatively weaker binding affinity.

Conversely, the interaction of SG-A with MLKL (PDB ID 6ZZ1) has a notable affinity, with a free binding energy of -9.40 kcal/mol, surpassing that of the co-crystallized ligand (-6.29 kcal/mol). This finding indicates that the superior binding efficiency of SG-A to the active site of MLKL is possibly facilitated by the wider configuration of the site [93], which seemingly accommodates SG-A more readily. Additionally, the active site of MLKL features a deeper pocket, likely enhancing the interaction strength between SG-A and essential amino acids and contributing to the observed increase in affinity. This differential affinity of SG-A for RIPK1, RIPK3, and MLKL indicates that while its structural rigidity may limit its binding to RIPK1 and RIPK3, it conversely benefits its interaction with MLKL due to its geometric compatibility with the protein’s active site.

**Fig 4:**
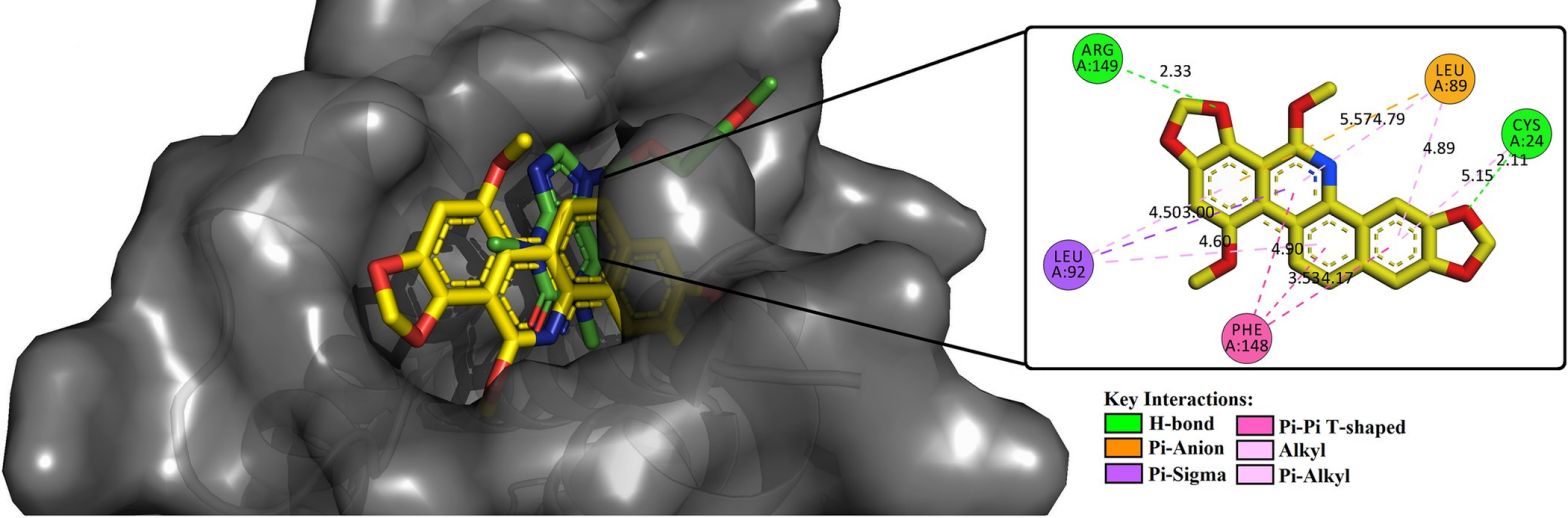


Fig 4 shows the molecular interactions of SG-A within the active site of MLKL. Notably, SG-A forms H-bonds with CYS24 and ARG149 at distances of 2.11 Å and 2.33 Å, respectively, alongside π-anion and π-sigma interactions with LEU89 and LEU92. The hydrophobic interactions with CYS24, LEU89, LEU92, and PHE148 further underscore the binding specificity of SG-A. These interactions mirror those of the co-crystallized ligand of MLKL (S3 Fig), indicating a comparable mode of binding and suggesting that the mechanistic pathway of SG-A induces necroptosis. While the interactions of SG-A with RIPK1 and RIPK3 revealed limited binding affinity and a lack of engagement with critical residues, as demonstrated in S4 and S5 Figs, respectively. SG-A exhibited a more favourable binding profile with MLKL, suggesting that, while SG-A may not serve as a potent inhibitor of RIPK1 or RIPK3, it holds significant potential in selectively targeting MLKL. This specificity underscores SG-A’s potential as a promising therapeutic candidate in necroptosis-driven cancer therapies, particularly for malignancies resistant to conventional apoptosis-inducing treatments. To further substantiate these findings, molecular dynamics (MD) simulations are warranted to evaluate the stability and dynamic behaviour of the SG-A-MLKL complex over time, thereby reinforcing SG-A’s potential as a targeted necroptosis inducer.

### 3.2 Molecular dynamics (MD) analysis

#### 3.2.1 Stability, PCA, and heat map analysis

MD simulations represent a pivotal advancement in drug discovery, enabling the elucidation of the temporal and spatial behaviours of molecular interactions within a biological system [94]. This computational technique offers a dynamic vista into the complex interplay between drug candidates and their target proteins, surpassing the static snapshots provided by molecular docking studies [95]. MD simulations are particularly invaluable for assessing the stability and flexibility of drug‒target complexes, which are key factors that significantly influence the pharmacodynamics and therapeutic efficacy of a compound [94]. In this study, leveraging insights gained from molecular docking, the optimal binding pose of SG-A within the active binding site of MLKL was selected for MD simulation over an extended period of 300 ns via GROMACS 2016. This selection was based on the optimal alignment and interaction profile of the pose with critical residues in the MLKL binding site, which is hypothesized to underpin the necroptotic induction capability of SG-A. Furthermore, to enable a comparative analysis, both the co-crystallized ligand and apo forms of MLKL were subjected to MD simulations. The stability of the docked complexes was analysed by calculating the root mean square deviation (RMSD), the root mean square fluctuation (RMSF) of the protein and ligand backbone atoms, and the radius of gyration (Rg). Prior to the simulation, the co-crystallized ligand (PDB ID: 6ZZ1) was removed from the apo form to serve as a starting structure for the simulation [50,51].

**Fig 5:**
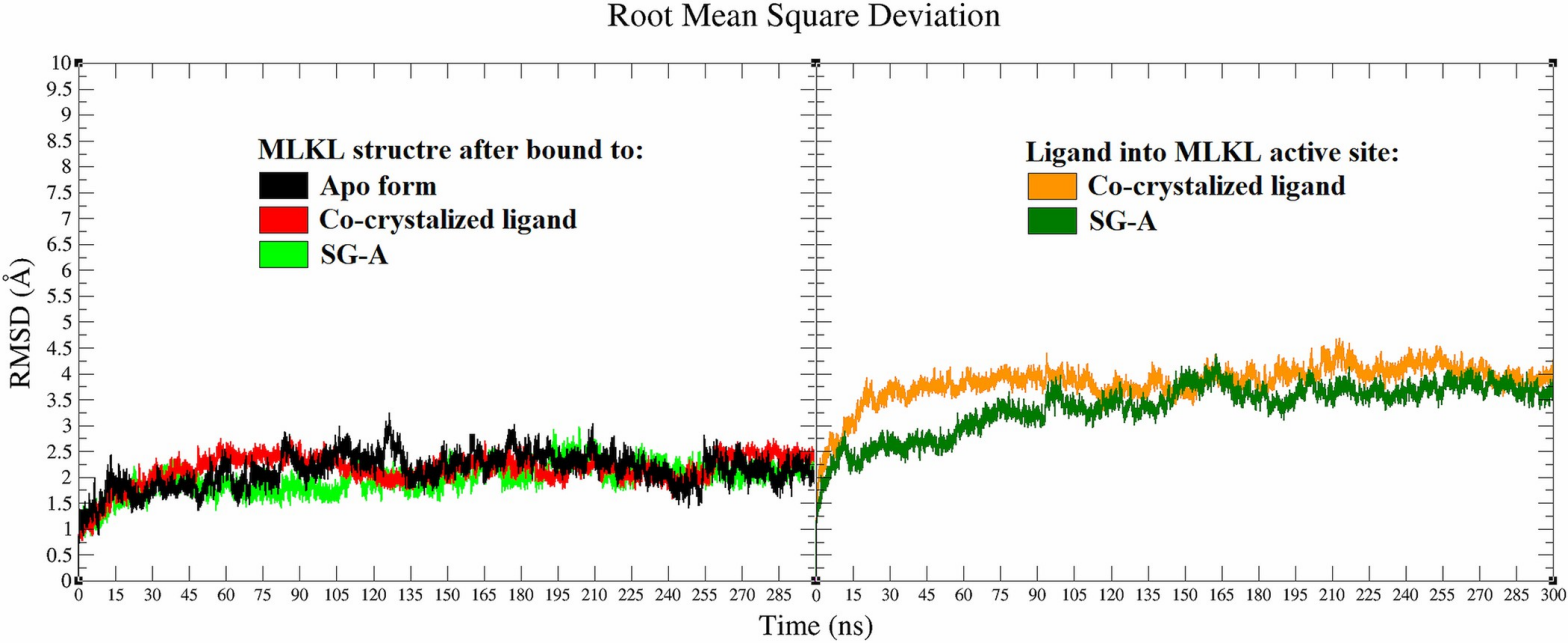


The RMSD of the protein backbone and ligand atoms was tracked throughout the MD simulations (300 ns) to assess the stability of the simulated systems [96]. Generally, stable RMSD values ranging from 1.4 to 3.3 Å were observed across all systems (protein backbones) during the simulation, achieving equilibrium after 135 ns (Fig 5). Upon comparing the initial and average structures following the MD simulations, a stable RMSD value of approximately 2.4 Å was observed for the protein in its apo form (black plot), reaching equilibrium after approximately 135 ns, with a slight deviation observed—increasing up to approximately 2.8 Å and decreasing to 1.4 Å, exhibiting a pattern of fluctuations similar on average until 300 ns (Fig 5). This observation closely aligns with the findings presented by Ramirez et al. [97]. For the MLKL protein backbone bound to the co-crystallized ligand (red), the average RMSD of the MLKL backbone fluctuated between 1.6 Å and 2.7 Å and remained consistent over the MD simulation period (300 ns), indicating a stable binding pose and consistent conformational changes. Notably, the MLKL protein backbone exhibited less deviation (more stability) upon binding with SG-A than did its apo and holo forms (bound to the co-crystallized ligand), with fluctuations ranging between 1.4 Å and 2.6 Å and continuing to fluctuate within these values until 300 ns. Interestingly, the deviation was less than 2 Å for the protein backbone in all systems, which falls within the acceptable range of deviation [44,51,55], indicating that SG-A did not adversely affect the protein backbone and potentially had a more favourable effect than the co-crystallized ligand did. The RMSD plot of SG-A showed lower RMSD deviations than those of the co-crystallized ligand throughout the 300 ns of the MD simulations. This finding could be attributed to the rigidity of the SG-A structure and its numerous interactions within the active binding site of MLKL, resulting in fewer conformational changes and a highly stable pose.

**Fig 6:**
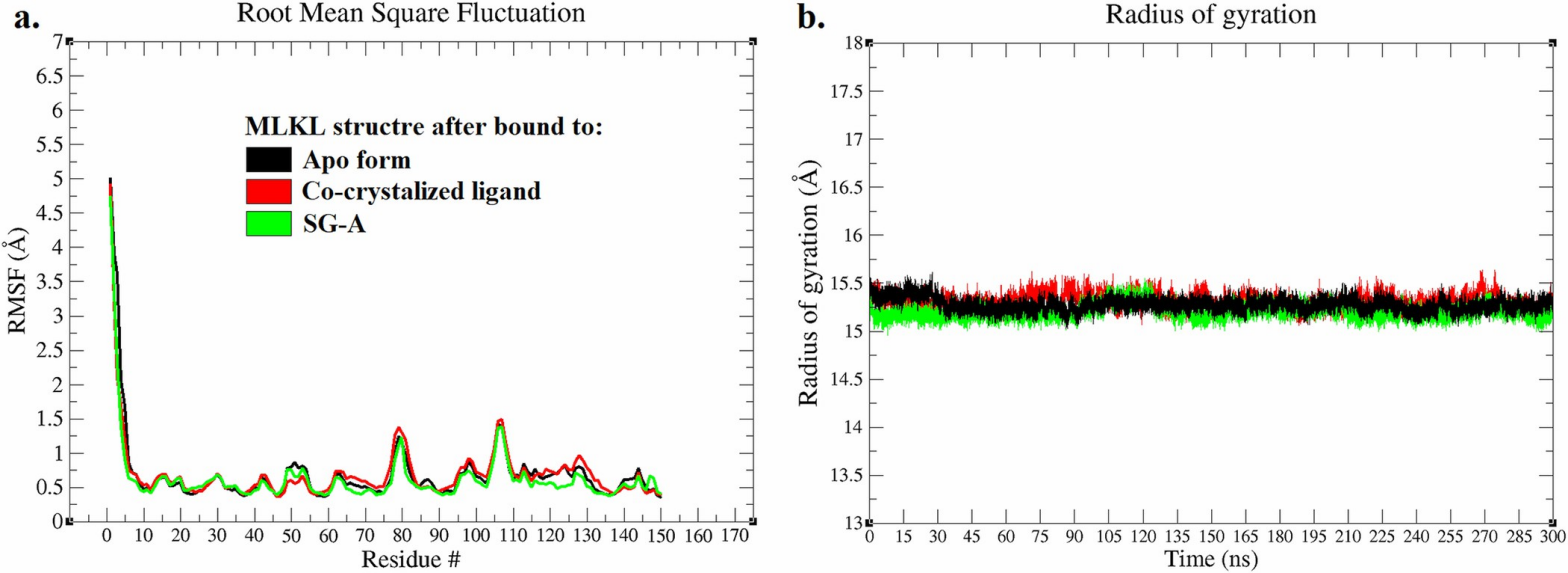


Following the RMSD analysis, the root-mean-square fluctuation (RMSF) was assessed for all systems throughout the 300 ns of the MD simulation, shedding light on the structural flexibility of the MLKL backbone atoms [44,55]. The average RMSF values were less than 1.7 Å for the MLKL backbone atoms across all the systems, except for the C-terminal residues (Fig 6A). Notably, no significant differences in RMSF values were observed between the apo and holo (co-crystallized ligand and SG-A) systems. These findings indicate that the structural integrity and stability of MLKL are maintained even in the presence of the lead molecule SG-A, suggesting that this molecule has a negative effect on protein flexibility. To perform the RMSF analysis, the compactness and size of the protein in both the apo and holo forms were gauged by calculating the Rg throughout the MD simulation timeframe of 0–300 ns [44,50,51]. The Rg results revealed a stable profile for all three systems, with a consistent value of approximately 15.3 Å (Fig 6B). This analysis further corroborates the findings from the RMSD and RMSF analyses, illustrating that SG-A does not compromise protein integrity and may indeed contribute to stabilizing the protein backbone.

After exploring the structure and size of our protein‒ligand systems through RMSD, RMSF, and Rg analyses, our study examined the hydrogen bond (H-bond) profile of the SG-A and MLKL complexes over the course of 300 ns MD simulations. The analysis of H-bonds, which are fundamental to the structural stability of biomolecular complexes [98], indicates the sustained interaction between SG-A and its target, MLKL. By assessing the H-bond profile, we aimed to highlight the stability of the SG-A-MLKL association, thus enhancing our understanding of the potential efficacy of SG-A.

**Fig 7:**
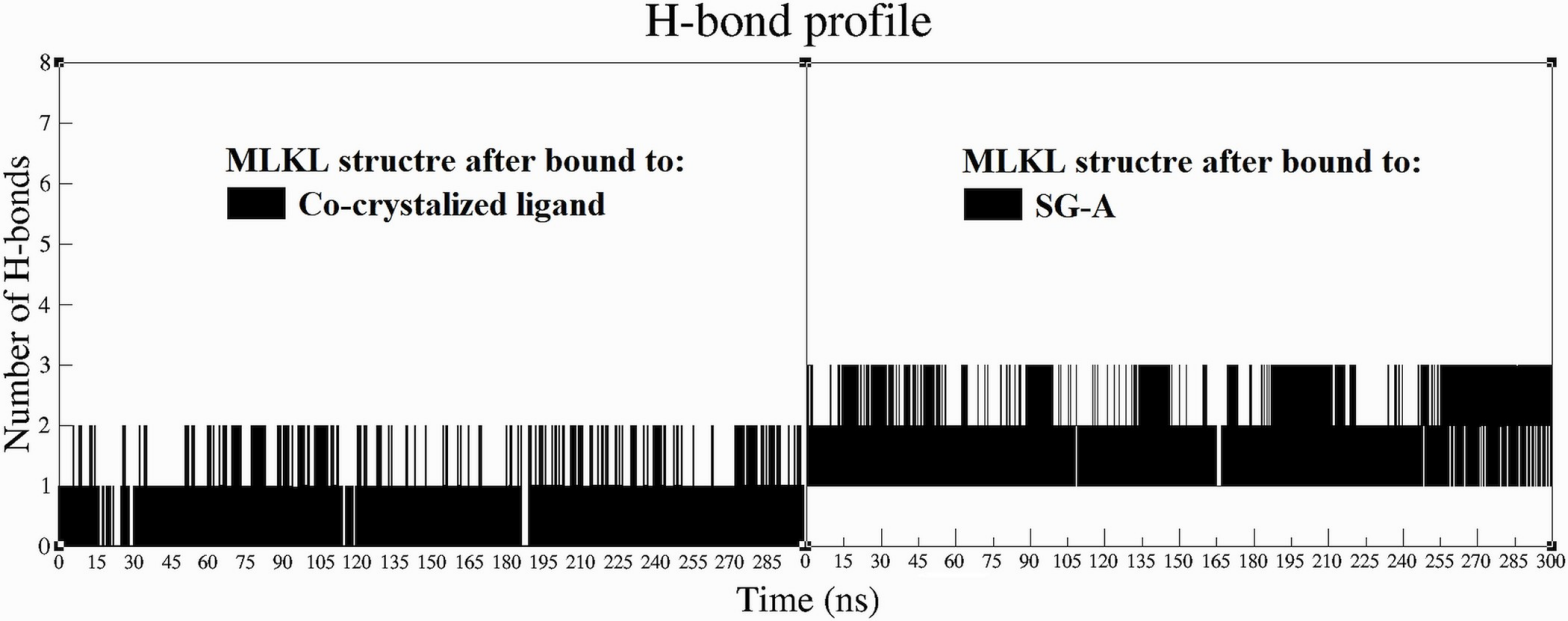


The analysis of H-bond profiles revealed notable interaction dynamics within the MLKL active binding site post-SG-A binding (Fig 7). Throughout the duration of the 300 ns MD simulations, SG-A demonstrated a remarkable ability to maintain two H-bonds with MLKL. This contrasts with the behavior of the co-crystallized ligand, which maintained only one H-bond over the same period (Fig 7). This observation is significant for several reasons. First, the sustained presence of two H-bonds between SG-A and MLKL suggested a greater degree of molecular affinity and interaction stability those of the co-crystallized ligand. This stability is crucial for the effectiveness of SG-A, as it implies more secure and enduring engagement with MLKL, potentially enhancing the necroptotic induction capabilities of the compound. Furthermore, the ability of SG-A to maintain multiple H-bonds indicates a well-suited conformational fit within the MLKL active site, which may contribute to its therapeutic potential.

**Fig 8:**
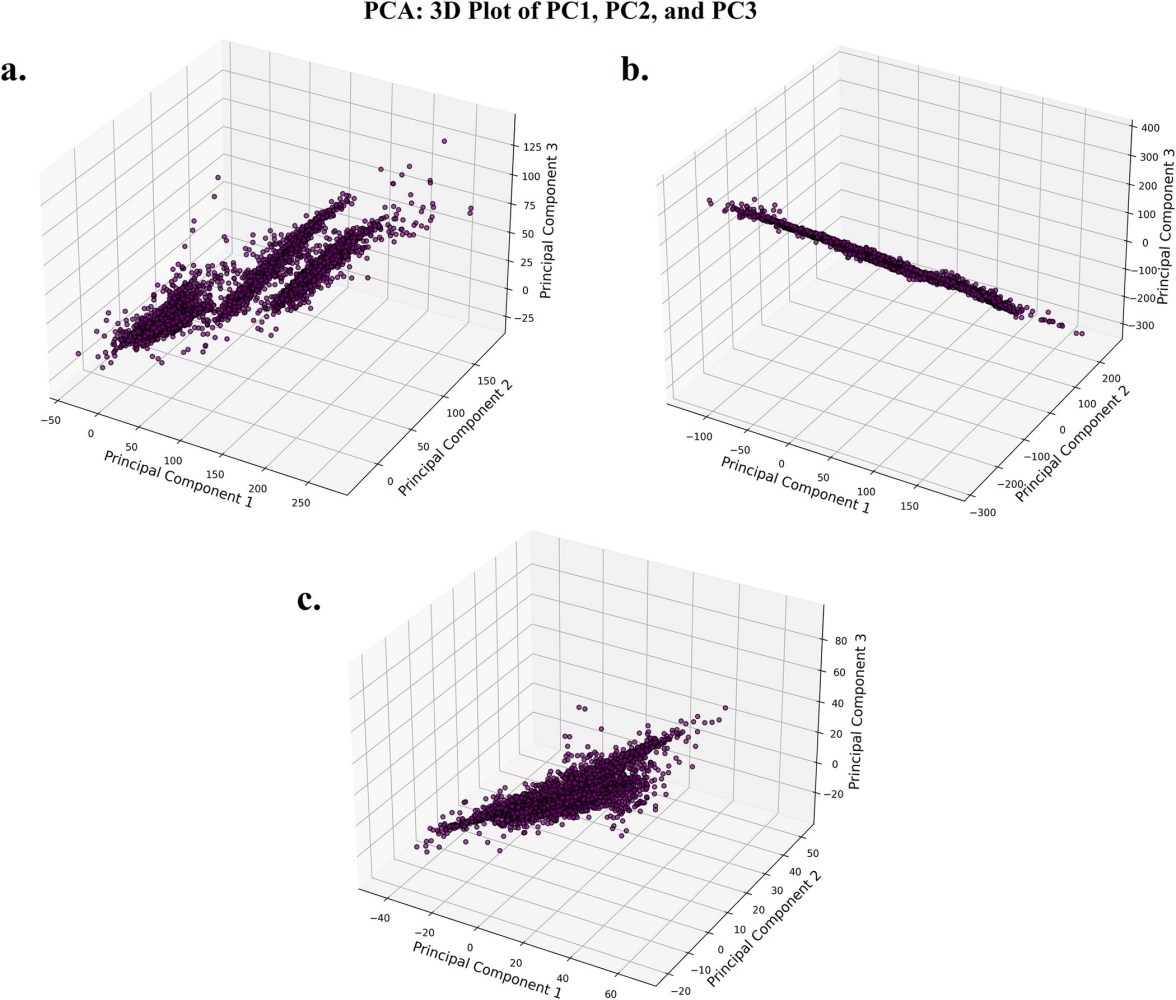


Following the hydrogen bond analysis, principal component analysis (PCA) was performed to investigate the conformational dynamics of MLKL via the interval time (0–300 ns) of MD simulations in three distinct states: the apo form, the form bound to the co-crystallized ligand, and the form bound to SG-A. PCA reduces the complex trajectory data from MD simulations into principal components (PCs) that describe the major collective motions of the protein [99]. The first three principal components (PC1, PC2, and PC3) were analysed to capture the most significant motions. In the apo form of MLKL (Fig 8A), the PCA plot reveals three distinct clusters, reflecting significant conformational diversity throughout the 300 ns simulation. This suggests that the unbound protein explores multiple conformational states, with the clusters representing distinct structural ensembles [100]. The wide separation between the clusters highlights the flexibility and adaptability of MLKL in its apo state [100]. Such conformational heterogeneity is often observed in proteins that are not bound to ligands, as they can adopt various configurations while searching for favourable binding partners [101].

For the MLKL bound to the co-crystallized ligand (Fig 8B), the PCA plot displays a more restricted distribution of data points, predominantly spread along a single plane. This implies that the protein adopts a more rigid conformation when bound to the ligand, with limited movement along PC2 and PC3. The confinement of the structure to this specific region of conformational space suggests that ligand binding stabilizes MLKL, reducing its flexibility [102]. Such stabilization is commonly observed in ligand-protein complexes, where binding often locks the protein into a specific functional conformation, limiting its structural rearrangement [103]. The SG-A-bound form of MLKL (Fig 8C) displays the most constrained behaviour, with the PCA plot showing a single, tight cluster of data points. This indicates that SG-A binding leads to a highly stable and rigid conformation, with minimal structural fluctuations throughout the 300 ns simulation. The dense clustering of points across all three principal components (PC1, PC2, and PC3) suggests that SG-A effectively "locks" MLKL into a specific conformation, further restricting its conformational space compared to the co-crystallized ligand [104]. This strong stabilizing effect implies that SG-A may have a significant impact on the protein’s functional dynamics by limiting its flexibility more effectively than the co-crystallized ligand.

**Fig 9:**
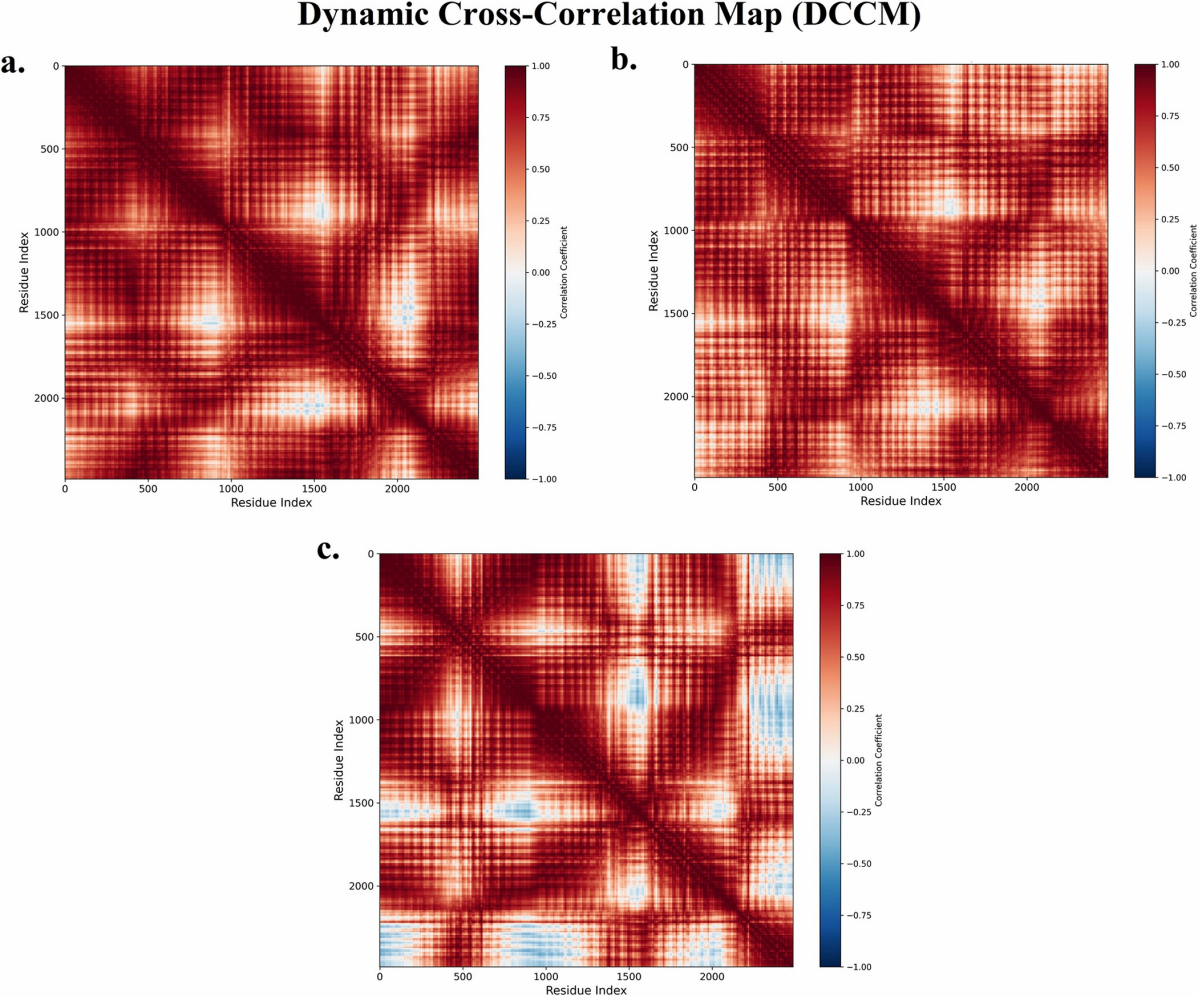


In this study, a dynamic cross-correlation map (DCCM) was also employed to evaluate the correlated motions of MLKL residues during the MD simulation [105]. The DCCM provides valuable insights into how different regions of the protein move relative to one another, highlighting both correlated (positive values) and anti-correlated (negative values) motions that may affect protein functionality [106,107]. By visualizing these motion patterns, the DCCM complements the PCA findings [107], offering a more detailed understanding of how SG-A and the co-crystallized ligand influence MLKL dynamics. In the apo form of MLKL (Fig 9A), the DCCM reveals several regions of highly correlated motions, as evidenced by the red clusters, along with dispersed anti-correlated regions (blue areas). This widespread distribution of both positive and negative correlations indicates that the protein undergoes significant internal motions, with different residue groups moving in concert or in opposition [106–108]. Such dynamic flexibility is characteristic of proteins in their unbound states, where they explore a broader conformational landscape, preparing for potential ligand binding [106–108].

For the MLKL bound to the co-crystallized ligand (Fig 9B), the DCCM shows a shift towards more uniform correlated motions, with fewer anti-correlated regions compared to the apo form. This suggests that ligand binding restricts the overall flexibility of MLKL, stabilizing specific regions and reducing the extent of opposing motions. The stabilization effect is consistent with the PCA results, where the protein displayed a more confined conformational space when bound to the co-crystallized ligand. The reduced flexibility observed here implies that the co-crystallized ligand locks MLKL into a functional conformation that supports its necroptotic signaling activity [109]. The most notable effect is observed in the DCCM for MLKL bound to SG-A (Fig 9C), where the cross-correlation map shows highly defined regions of correlated motions, with significantly fewer anti-correlated areas. This suggests that SG-A binding induces a marked stabilization of the protein, minimizing the internal dynamic fluctuations between residues [106–108]. The strong positive correlations seen across key regions of MLKL imply that SG-A enforces a rigid, stable conformation, further supporting the notion that SG-A locks MLKL into a specific functional state [106–108]. This high level of stabilization could enhance SG-A’s necroptotic potential by ensuring that MLKL remains in an active conformation, optimizing its role in the necroptosis pathway.

#### 3.2.2 MM-PBSA and individual residue contribution analysis

Given the demonstrated consistency and stability within the 270–300 ns region, as evidenced by the RMSD analyses (Fig 5) and simulation time plots, the coordinates obtained from the production phase of this interval were employed to calculate the free binding energies of the simulated systems. To quantify the interaction strength between SG-A and MLKL, the Molecular Mechanics Poisson-Boltzmann Surface Area (MM-PBSA) method was utilized. MM-PBSA facilitates post-simulation analysis by estimating the free energy of binding, taking into account contributions from molecular mechanics, solvation effects, and entropy variations [72,73,110]. This method is instrumental in providing a more detailed assessment of binding affinity, offering insights that extend beyond conventional metrics [72,73,110].

By analysing the data presented in Table 2, we observed the MM-PBSA binding energies (Δ*G*_bind_) of SG-A in comparison to those of the co-crystallized ligand (control) at the MLKL active site (PDB ID: 6ZZ1) during the specified MD interval time (270–300 ns). The binding energy of SG-A to MLKL was significantly greater (−31.03 ± 0.16 kcal/mol) than that of the control (−23.96 ± 0.11 kcal/mol), indicating a greater affinity of SG-A for the MLKL receptor. The major contributors to this binding energy are worth noting. The electrostatic and van der Waals interactions of SG-A with MLKL were significantly stronger (more negative) than those of the control, as indicated by the electrostatic (−13.16 ± 0.18 kcal/mol for SG-A vs. −9.64 ± 0.17 kcal/mol for the control) and van der Waals (−17.43 ± 0.19 kcal/mol for SG-A vs. −14.35 ± 0.20 kcal/mol for the control) energy components. These findings suggest that SG-A forms a more tightly bound complex with MLKL, potentially enhancing its efficacy as a necroptosis inducer [111].

**Table 2:** MM-PBSA binding energies (Δ*G*_bind_) of SG-A and the co-crystalized ligand (control) at the active site of MLKL (PDB ID: 6ZZ1) for the MD interval time (270–300 ns). Energy Units are Expressed in kcal/mol.

System	Δ*G*_bind_ (kcal/mol)	Electrostatic (kcal/mol)	Van der Waal (kcal/mol)	Polar Salvation (kcal/mol)	Non-Polar Salvation (kcal/mol)
**MLKL-SG-A**	−31.03 ± 0.16	−13.16 ± 0.18	−17.43 ± 0.19	11.41 ± 0.13	−11.85 ± 0.14
**MLKL-Control**	−23.96 ± 0.11	−9.64 ± 0.17	−14.35 ± 0.20	10.87 ± 0.12	−10.84 ± 0.13

Interestingly, the polar solvation energy, although unfavourable for both SG-A and the control, is slightly more unfavourable for SG-A (11.41 ± 0.13 kcal/mol for SG-A vs. 10.87 ± 0.12 kcal/mol for the control), possibly because of its stronger interaction with the protein, which may lead to a more significant rearrangement of the water molecules in the binding site [112]. Conversely, the nonpolar solvation component, which contributes favourably to the binding energy, is more negatively related to SG-A (−11.85 ± 0.14 kcal/mol) than to the control (−10.84 ± 0.13 kcal/mol), reflecting the hydrophobic contributions to the stability of the SG-A-MLKL complex [113]. The individual contributions of key amino acid residues to the binding of SG-A and the co-crystallized ligand within MLKL, as presented in Table 3, provide further insights into the interaction dynamics. Notably, residues such as CYS24, LEU89, LEU92, and ARG149 exhibit significant energy contributions, particularly in the MLKL-SG-A complex. The interaction of SG-A with ARG149, for instance, contributes −176.24 kcal/mol, compared to −174.87 kcal/mol for the control ligand, underscoring the stronger affinity of SG-A for MLKL. Similarly, LEU89 and LEU92 show a little bit greater binding energy (more negative) for SG-A than the control, suggesting that hydrophobic interactions play a critical role in stabilizing the SG-A-MLKL complex.

**Table 3:** The contribution of individual amino acid residues (key residues) of MLKL for SG-A and the co-crystalized ligand (control) for the MD interval time (270–300 ns). Energy Units are Expressed in kcal/mol.

System	CYS24	ILE85	LEU89	LEU92	PHE148	ARG149
**MLKL-SG-A**	-15.05	-7.62	-17.47	-11.04	-11.41	-176.24
**MLKL-Control**	-14.08	-8.76	-16.50	-9.84	-12.09	-174.87

These residue-level contributions support the overall findings from the MM-PBSA analysis, suggesting that SG-A may have similar or even greater binding affinity for MLKL compared to the co-crystallized ligand. The comparable or stronger interactions of key residues, such as ARG149, LEU89, and LEU92, highlight SG-A’s potential to effectively modulate the necroptosis pathway. To further elucidate the electronic stability of the SG-A-MLKL complex and provide a deeper understanding of the interaction mechanisms at play, density functional theory (DFT) calculations were performed. DFT analysis allows for the exploration of electronic properties and stability, complementing the binding affinity data by offering insights into the electronic configurations that underlie the strong interaction between SG-A and MLKL.

### 3.3 Molecular reactivity analysis

#### 3.3.1 Density functional theory (DFT)

**Fig 10:**
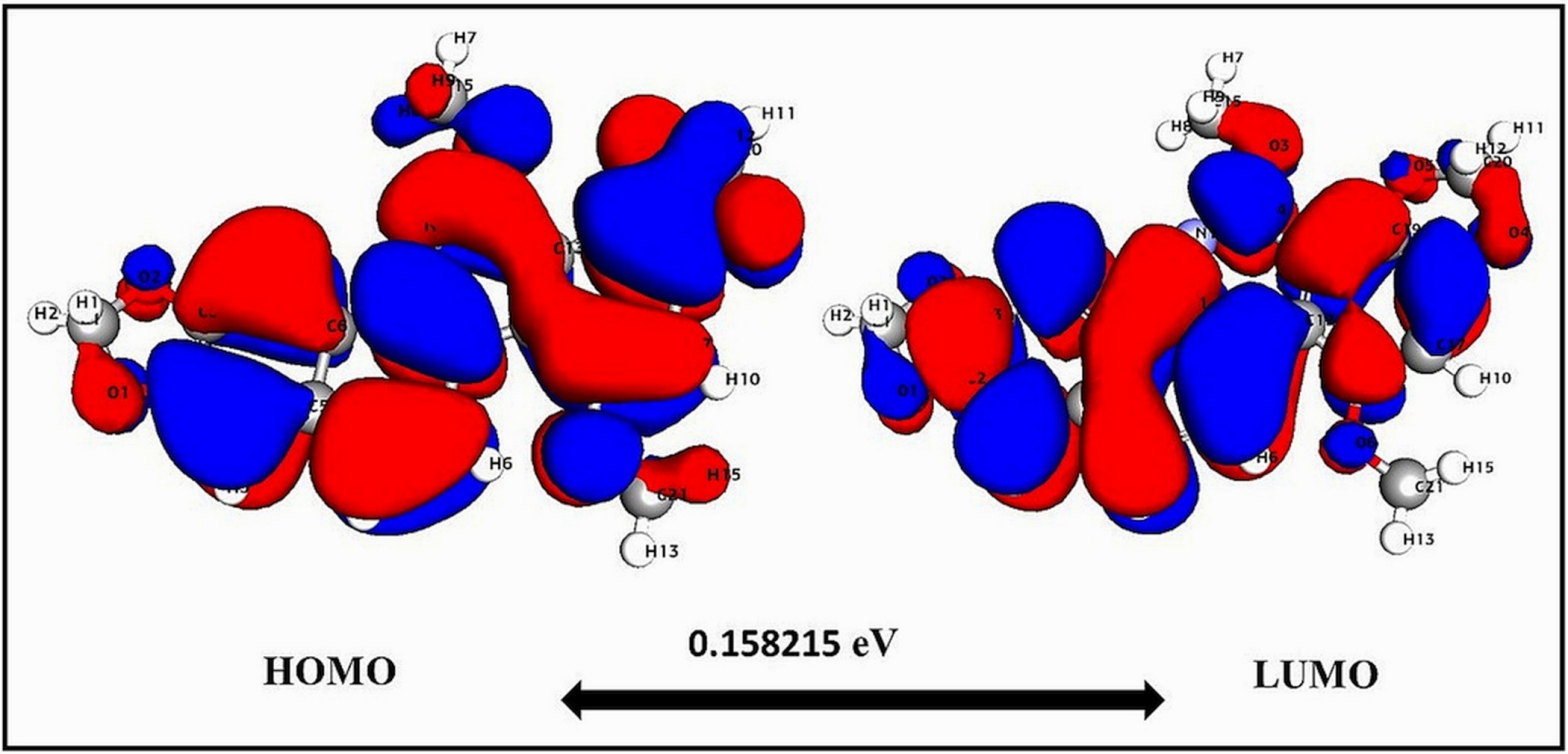


DFT calculations for SG-A, as presented in S2 Table, revealed critical aspects of its electronic structure and reactivity [114], which are essential for understanding its interactions with the MLKL receptor and its efficacy as a necroptosis inducer. The total energy of the molecule, recorded at -1393.5 units, along with a binding energy of -90.38 units, indicates that SG-A is highly stable and has a significant affinity for MLKL. This strong binding energy underlines the potential of SG-A to form stable complexes, which are crucial for its ability to induce necroptosis [48]. Further analysis of the highest occupied molecular orbital (HOMO) and lowest unoccupied molecular orbital (LUMO) energies of SG-A revealed values of -0.22 and -0.06 units, respectively, resulting in a notably narrow band gap of ~0.16 units (Fig 10). This characteristic suggests that the propensity for SG-A to participate in electron transfer processes is vital for its effective binding to MLKL, which is indicative of a molecule with high reactivity and low kinetic stability that is prone to biological interactions [48].

Moreover, the dipole moment of 2.64 Debye indicates an asymmetric charge distribution within SG-A, potentially enhancing its solubility and interaction within the polar biological milieu [115]. A molecular hardness (η) of 0.15 and a softness (S) of 6.32 further denote the high reactivity and biological activity potential of SG-A, given that greater softness typically correlates with enhanced activity [116]. SG-A’s electrophilicity index (ω) of 22.16 portrays it as a strong electron acceptor, suggesting that its aggressive electrophilic nature is crucial for binding to nucleophilic sites on the MLKL protein [48]. Concurrently, the slight electronegativity of -0.13 emphasizes its capacity for electrostatic interactions, which are fundamental for initial docking and stable binding with MLKL [117].

Building on the foundational insights from the DFT calculations, we performed a molecular electrostatic potential (MEP) analysis. This next phase of investigation aims to elaborate on the charge distribution across SG-A, providing a deeper understanding of its interaction dynamics. By transitioning to MEP analysis, we anticipate further elucidating the comprehensive role of SG-A as an inducer of necroptosis, augmenting our understanding of its mechanistic profile and reinforcing its therapeutic potential.

#### 3.2.2 Molecular electrostatic potential (MEP) analysis

**Fig 11:**
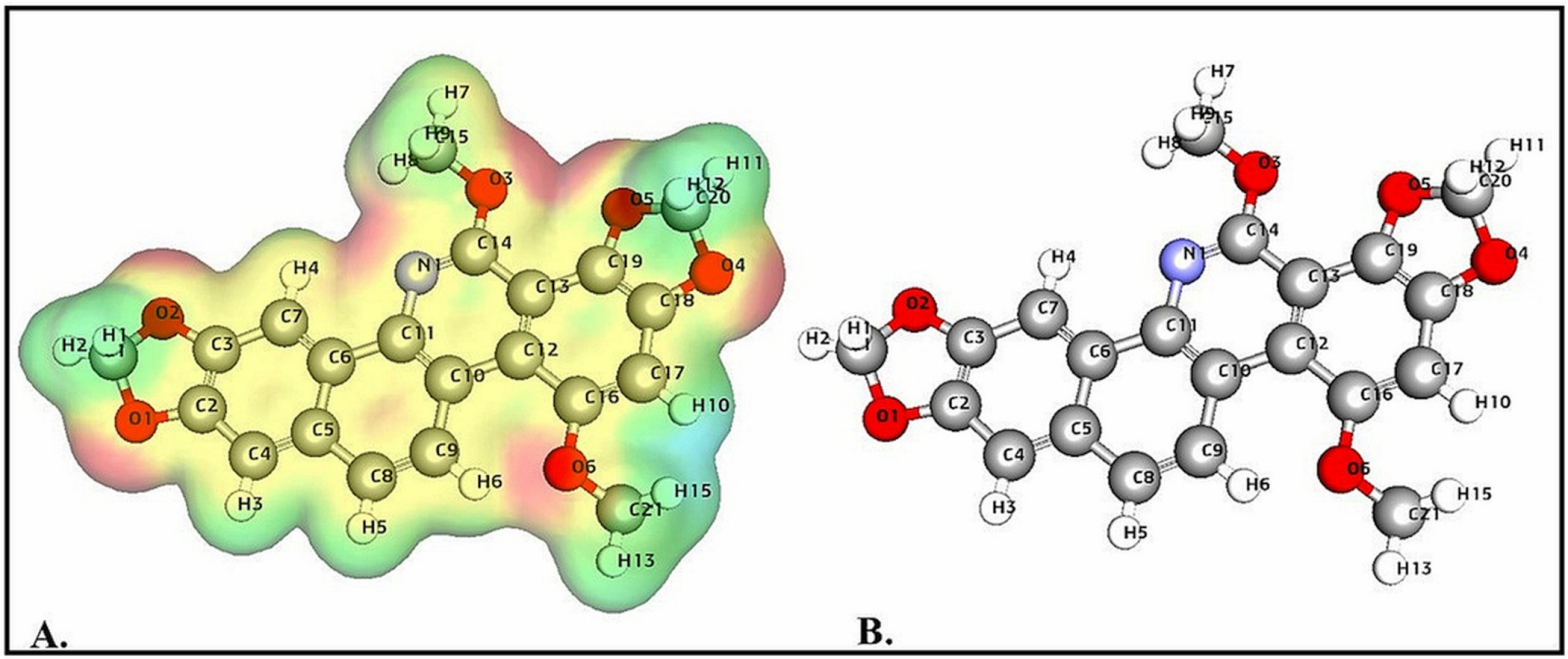


MEP analysis is a pivotal tool for identifying molecular regions susceptible to electrophilic attack, nucleophilic reactivity, and hydrogen bonding interactions [118]. By utilizing color-coded schemes, MEP analysis facilitates the visualization of a molecule’s structural attributes in tandem with its electrostatic potentials, effectively highlighting positive, negative, and neutral regions. Specifically, Fig 11 presents the MEP analysis for SG-A, which offers vital insights into its electrostatic properties. MEPs provide a three-dimensional visualization of the electrostatic field encompassing a molecule by calculating the electrostatic potential at various points around the molecule, which is introduced by a point charge [48]. This methodology is crucial for understanding and predicting a range of chemical properties, including reactivity, binding affinity, and pKa. The distribution of electrostatic potentials within SG-A may provide insight into its interaction behaviours and chemical reactivity in various environments.

In SG-A, the alternating regions of positive and negative charge are notably influenced by the electron distribution within the molecule, which, in turn, is dictated by the electronegativity variations among its constituent atoms. The oxygen atoms (O1, O2, O4, O6) and the central nitrogen atom (N1) are more electronegative, attracting electrons and creating regions of negative electrostatic potential. In contrast, hydrogen atoms (H3—H15) are associated with a positive electrostatic potential. Although a precise determination of the overall electrostatic potential of SG-A via visual analysis might be challenging, the balanced distribution of charges suggests an approximate net zero, which is indicative of a molecule with balanced electrophilic and nucleophilic regions. The methoxy groups attached to the benzene rings significantly influence the molecule’s electrostatic potential, with oxygen atoms drawing electrons towards themselves, thereby creating negative electrostatic zones. Furthermore, the central nitrogen atom (N1) is expected to increase the overall negative potential of the molecule, which is crucial for its biological interactions.

Integrating these MEP insights with the DFT findings highlights the potential of SG-A as a necroptosis inducer, complementing and supporting molecular dynamics (MD) analysis outcomes. The detailed electrostatic characterization, revealing a balanced distribution of charge and areas of specific reactivity, aligns with the MD analysis, which demonstrated the strong and stable interaction of SG-A with the MLKL receptor. This harmony between the computational analyses highlights the robust molecular basis for the efficacy of SG-A in biological systems, suggesting its significant potential for inducing necroptosis. Although the findings presented in this study are promising, they are derived solely from theoretical and computational approaches, including molecular docking, molecular dynamics simulations, DFT, and MEP analyses. While these methods provide valuable predictive insights, experimental validation is crucial to substantiate the conclusions. In particular, *in vitro* and *in vivo* studies are necessary to confirm the necroptotic potential of SG-A, as well as to assess its pharmacokinetics, bioavailability, and therapeutic efficacy.

## 4. Conclusions

This comprehensive *in silico* study highlights the potential of 8,12-dimethoxysanguinarine (SG-A) as a promising therapeutic agent for breast cancer through the induction of necroptosis. Our molecular docking, MD simulations (300 ns), DFT, and MEP analyses revealed enhanced binding affinity and stability of SG-A with the MLKL protein, demonstrated by a notable binding energy of -9.40 kcal/mol, surpassing that of the co-crystallized ligand (-6.29 kcal/mol). Although SG-A exhibited moderate affinity for RIPK3 (-7.01 kcal/mol) and RIPK1 (-6.37 kcal/mol), its interaction with MLKL showed the most potential. MD simulations confirmed the stability of SG-A within the MLKL active site, with RMSD values stabilizing between 1.4 and 3.3 Å, while RMSF analysis indicated that protein backbone flexibility was maintained. The formation of two key hydrogen bonds with MLKL further underscored the robust interaction dynamics of SG-A. In addition, MM-PBSA analyses supported the binding efficacy of SG-A, with a binding free energy of -31.03 ± 0.16 kcal/mol, surpassing the control ligand. Individual residue contributions, particularly ARG149 (-176.24 kcal/mol), further emphasized the strong interaction of SG-A within the MLKL binding site. The PCA analysis revealed that SG-A binding significantly reduced the conformational space of MLKL, indicating enhanced stabilization, while the DCCM analysis demonstrated highly correlated residue motions, reinforcing the stability of the SG-A-MLKL complex. DFT and MEP analyses provided further support for SG-A’s stable binding interactions, characterized by favourable electronic properties, including a narrow band gap and an electrostatic potential conducive to necroptosis induction. Despite these promising findings, this study’s reliance on computational methods requires experimental validation to fully realize SG-A’s therapeutic potential. Future *in vitro* and *in vivo* studies are necessary to confirm its efficacy, pharmacokinetics, pharmacodynamics, and safety profile. Furthermore, detailed investigations into the mechanisms by which SG-A induces necroptosis will be critical for its optimization as a cancer therapeutic. Expanding this research to other cancer cell lines, including those resistant to conventional therapies, may further elucidate SG-A’s broader therapeutic applicability.

## Supporting information

S1 Table Grid box coordinates of protein-ligand interactions as determined by co-crystallized ligands. (DOCX)

S2 Table Density function theory (DFT) calculations with other descriptors. (DOCX)

S1 Fig Superimposition (a) and 2D interactions analysis of the co-crystallized ligand (green C, red O, and blue N) (b) and re-docked ligand (lime C, red O, and blue N) (c). The crystal structure of human RIPK1 kinase domain in complex with GNE684 (6NYH.pdb) (RMSD is 0.67 Å).
(TIF)

S2 Fig Superimposition (a) and 2D interactions analysis of the co-crystallized ligand (green C, red O, and blue N) (b) and re-docked ligand (lime C, red O, and blue N) (c). The crystal structure of human RIPK3 in complex with N-[4-((2-[(cyclopropanecarbonyl)amino]pyridin-4-yl)oxy)-3-fluorophenyl]-1-(4-fluorophenyl)-2-oxo-1,2-dihydropyridine-3-carboxamide (7MON.pdb) (RMSD is 1.22 Å).
(TIF)

S3 Fig Superimposition (a) and 2D interactions analysis of the co-crystallized ligand (green C, red O, and blue N) (b) and re-docked ligand (lime C, red O, and blue N) (c). Human crystal structure of MLKL executioner domain in complex with a covalent inhibitor (7-(2-methoxyethoxymethyl)-1,3-dimethyl-purine-2,6-dione)) (6ZZ1.pdb) (RMSD is 1.13Å).
(TIF)

S4 Fig Superimposition of SG-A and the co-crystallized ligand within the active binding site of RIPK1 (a), along with 2D molecular interaction analysis (b). The co-crystallized ligand is shown with carbon atoms in green, oxygen in red, and nitrogen in blue, while SG-A is represented with carbon atoms in yellow, oxygen in red, and nitrogen in blue.
(TIF)

S5 Fig Superimposition of SG-A and the co-crystallized ligand within the active binding site of RIPK3, along with 2D molecular interaction analysis. The co-crystallized ligand is shown with carbon atoms in green, oxygen in red, and nitrogen in blue, while SG-A is represented with carbon atoms in yellow, oxygen in red, and nitrogen in blue.
(TIF)
