# Expression of Concern: Targeting necroptosis in MCF-7 breast cancer cells: *In Silico* insights into 8,12-dimethoxysanguinarine from *Eomecon Chionantha* through molecular docking, dynamics, DFT, and MEP studies

**DOI:** 10.1371/journal.pone.0347090

**Published:** 2026-04-13

**Authors:** The PLOS One Editors

After this article [1] was published, concerns were noted about the following:

- Compliance with the PLOS Authorship policy;
- The article cited as Reference 81 was retracted after [1] was published.

In light of the cumulative, the *PLOS One* Editors issue this Expression of Concern. Readers are advised to interpret the article [1] with caution.
