# Supplementary material for: Targeting necroptosis in MCF-7 breast cancer cells: In Silico insights into 8,12-dimethoxysanguinarine from Eomecon Chionantha through molecular docking, dynamics, DFT, and MEP studies
Source: PLoS One. 2025 Jan 7;20(1):e0313094. doi: 10.1371/journal.pone.0313094 (PMC11706375; doi:10.1371/journal.pone.0313094)
Supplement: S1 Table — (DOCX) [file pone.0313094.s001.docx]

**Supplementary Materials**

| **Table S1.** Grid box coordinates of protein-ligand interactions as determined by co-crystallized ligands. | | | | |
| --- | --- | --- | --- | --- |
| Crystal structure | Grid box size | Active binding sites coordinate (xyz-coordinates) | | |
|  |  | X | Y | Z |
| RIPK1-6NYH | 40*40*40 | -9.255 | -7.071 | 56.204 |
| RIPK3-6NYH | 40*40*40 | -40.564 | 2.879 | -19.703 |
| MLKL-6ZZ1 | 40*40*40 | 14.177 | -9.481 | -3.263 |
