# Supplementary material for: Targeting necroptosis in MCF-7 breast cancer cells: In Silico insights into 8,12-dimethoxysanguinarine from Eomecon Chionantha through molecular docking, dynamics, DFT, and MEP studies
Source: PLoS One. 2025 Jan 7;20(1):e0313094. doi: 10.1371/journal.pone.0313094 (PMC11706375; doi:10.1371/journal.pone.0313094)
Supplement: S2 Table — (DOCX) [file pone.0313094.s002.docx]

| **Table S2.** Density function theory (DFT) calculations with other descriptors. | | | | | | | | | | |
| --- | --- | --- | --- | --- | --- | --- | --- | --- | --- | --- |
| **Name** | **Total energy** | **Binding Energy** | **HOMO Energy** | **LUMO Energy** | **Band Gap Energy** | **Dipole moment** | **Hardness**  **(η)** | **Softness**  **(S)** | **Electro-**  **philicity (ω)** | **Electroneg-ativity (χ)** |
| **SG-A** | -1393.5 | -90.38 | -0.22 | -0.06 | 0.16 | 2.64 | 0.15 | 6.32 | 22.16 | -0.13 |
