# Supplementary figures and images for: Targeting necroptosis in MCF-7 breast cancer cells: In Silico insights into 8,12-dimethoxysanguinarine from Eomecon Chionantha through molecular docking, dynamics, DFT, and MEP studies
Source: PLoS One. 2025 Jan 7;20(1):e0313094. doi: 10.1371/journal.pone.0313094 (PMC11706375; doi:10.1371/journal.pone.0313094)

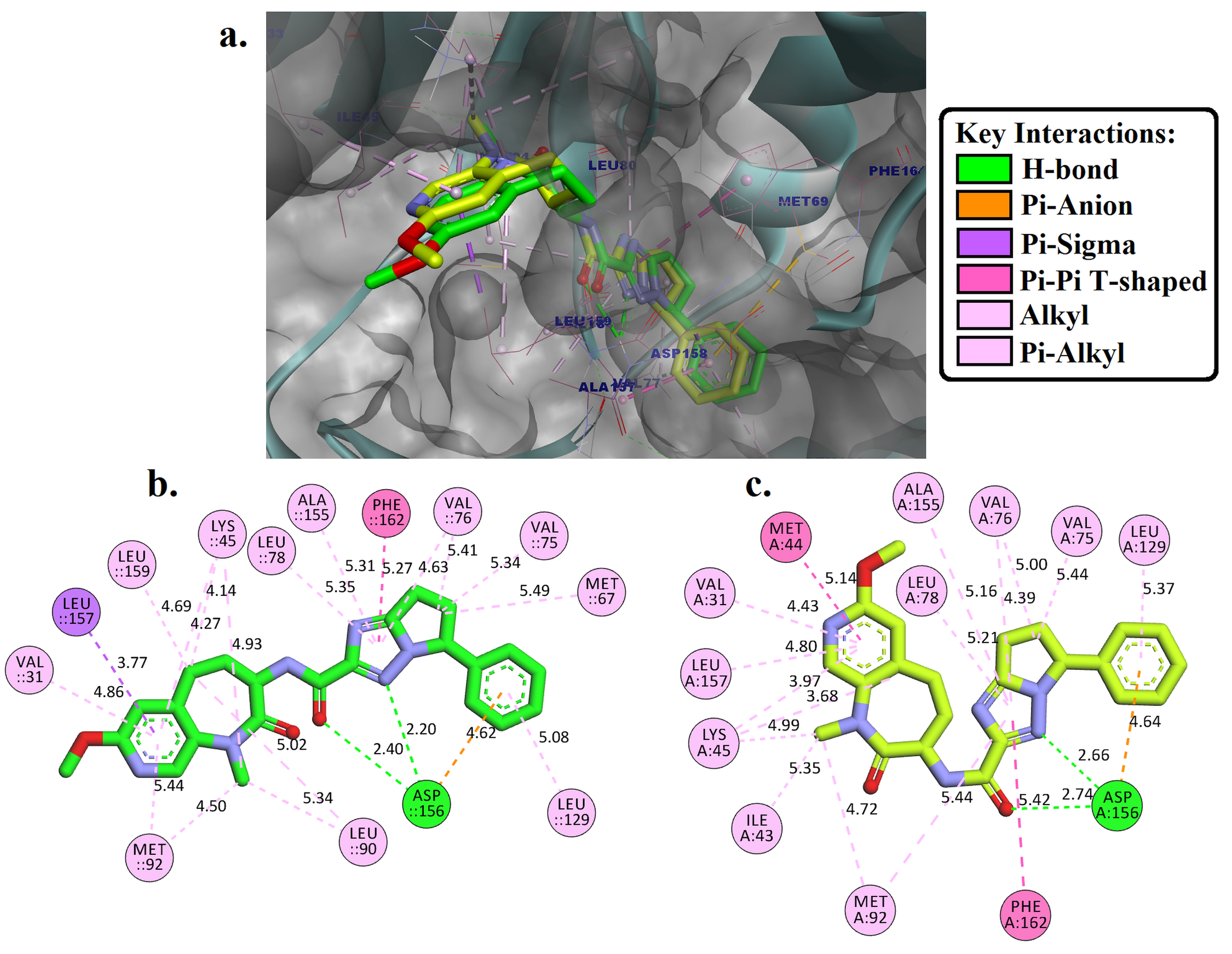

Supplement: S1 Fig — Superimposition (a) and 2D interactions analysis of the co-crystallized ligand (green C, red O, and blue N) (b) and re-docked ligand (lime C, red O, and blue N) (c). The crystal structure of human RIPK1 kinase domain in complex with GNE684 (6NYH.pdb) (RMSD is 0.67 Å). (TIF) [file pone.0313094.s003.tif]

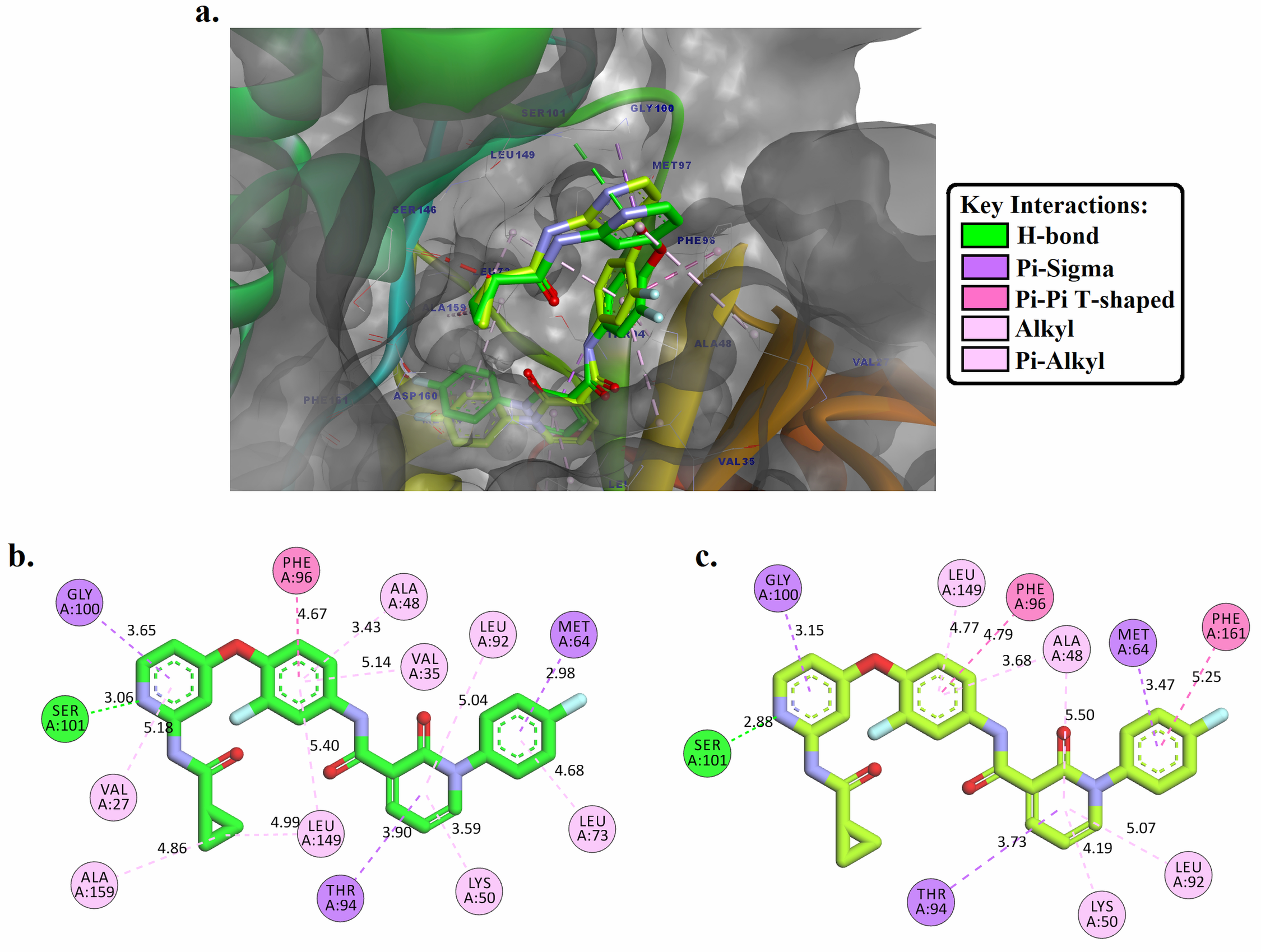

Supplement: S2 Fig — Superimposition (a) and 2D interactions analysis of the co-crystallized ligand (green C, red O, and blue N) (b) and re-docked ligand (lime C, red O, and blue N) (c). The crystal structure of human RIPK3 in complex with N-[4-((2-[(cyclopropanecarbonyl)amino]pyridin-4-yl)oxy)-3-fluorophenyl]-1-(4-fluorophenyl)-2-oxo-1,2-dihydropyridine-3-carboxamide (7MON.pdb) (RMSD is 1.22 Å). (TIF) [file pone.0313094.s004.tif]

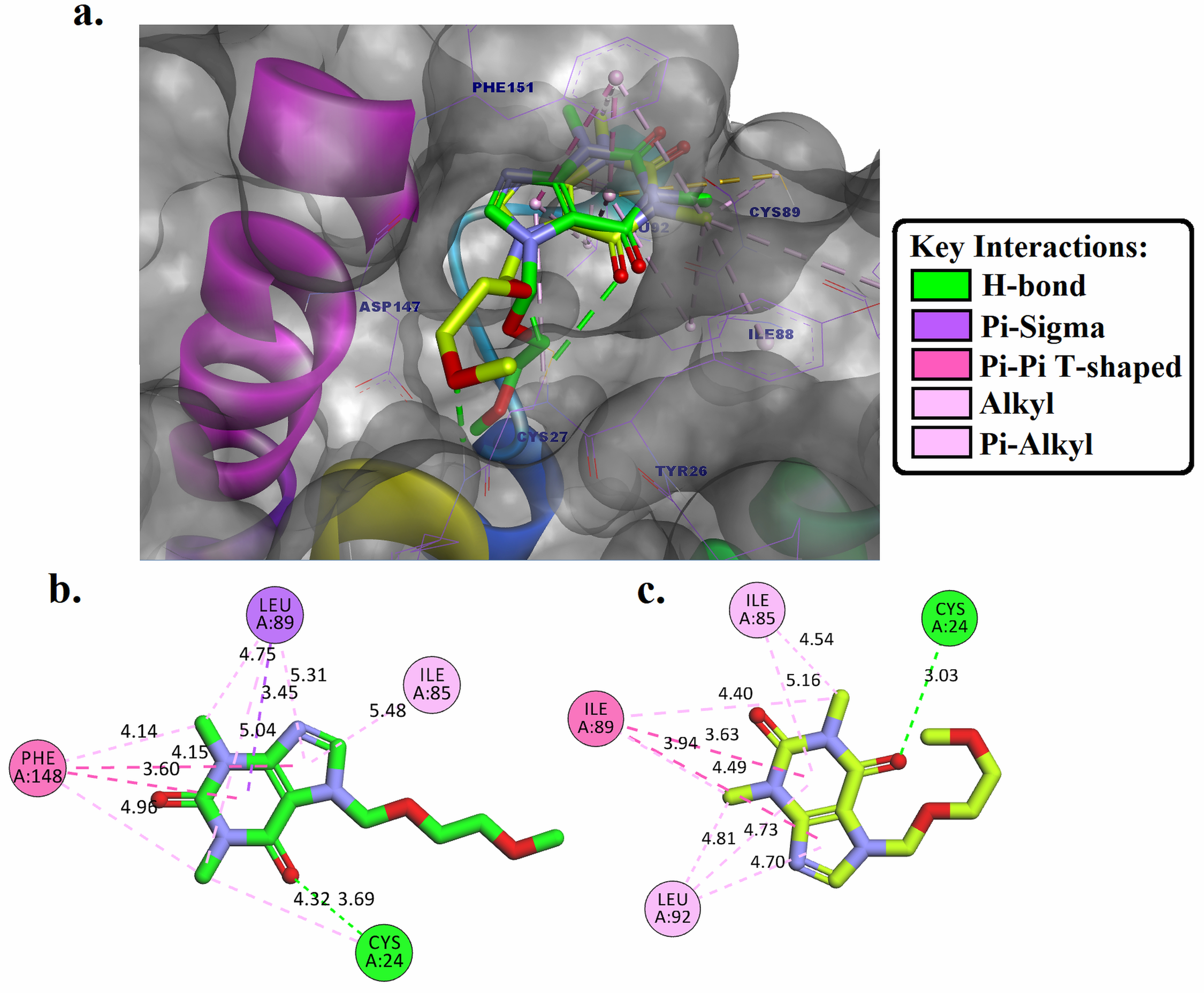

Supplement: S3 Fig — Superimposition (a) and 2D interactions analysis of the co-crystallized ligand (green C, red O, and blue N) (b) and re-docked ligand (lime C, red O, and blue N) (c). Human crystal structure of MLKL executioner domain in complex with a covalent inhibitor (7-(2-methoxyethoxymethyl)-1,3-dimethyl-purine-2,6-dione)) (6ZZ1.pdb) (RMSD is 1.13Å). (TIF) [file pone.0313094.s005.tif]

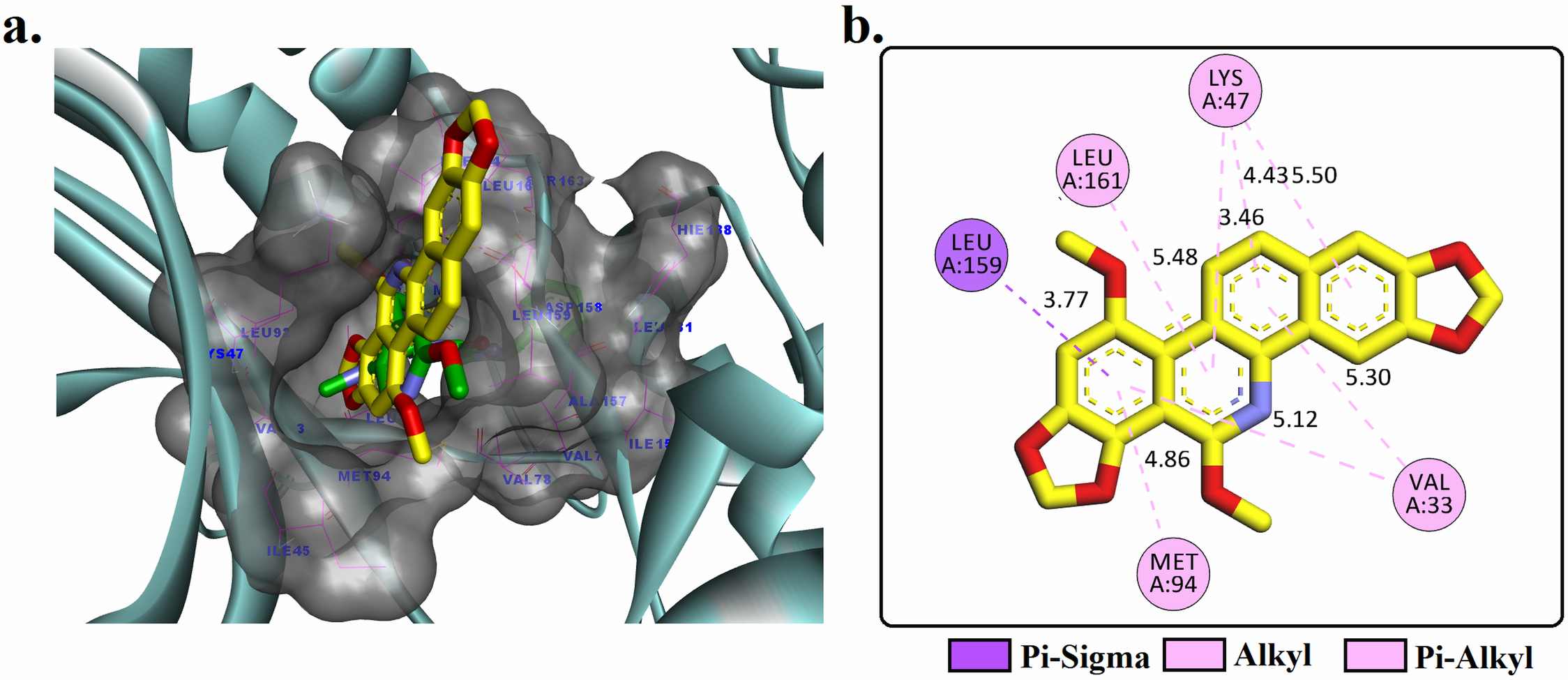

Supplement: S4 Fig — Superimposition of SG-A and the co-crystallized ligand within the active binding site of RIPK1 (a), along with 2D molecular interaction analysis (b). The co-crystallized ligand is shown with carbon atoms in green, oxygen in red, and nitrogen in blue, while SG-A is represented with carbon atoms in yellow, oxygen in red, and nitrogen in blue. (TIF) [file pone.0313094.s006.tif]

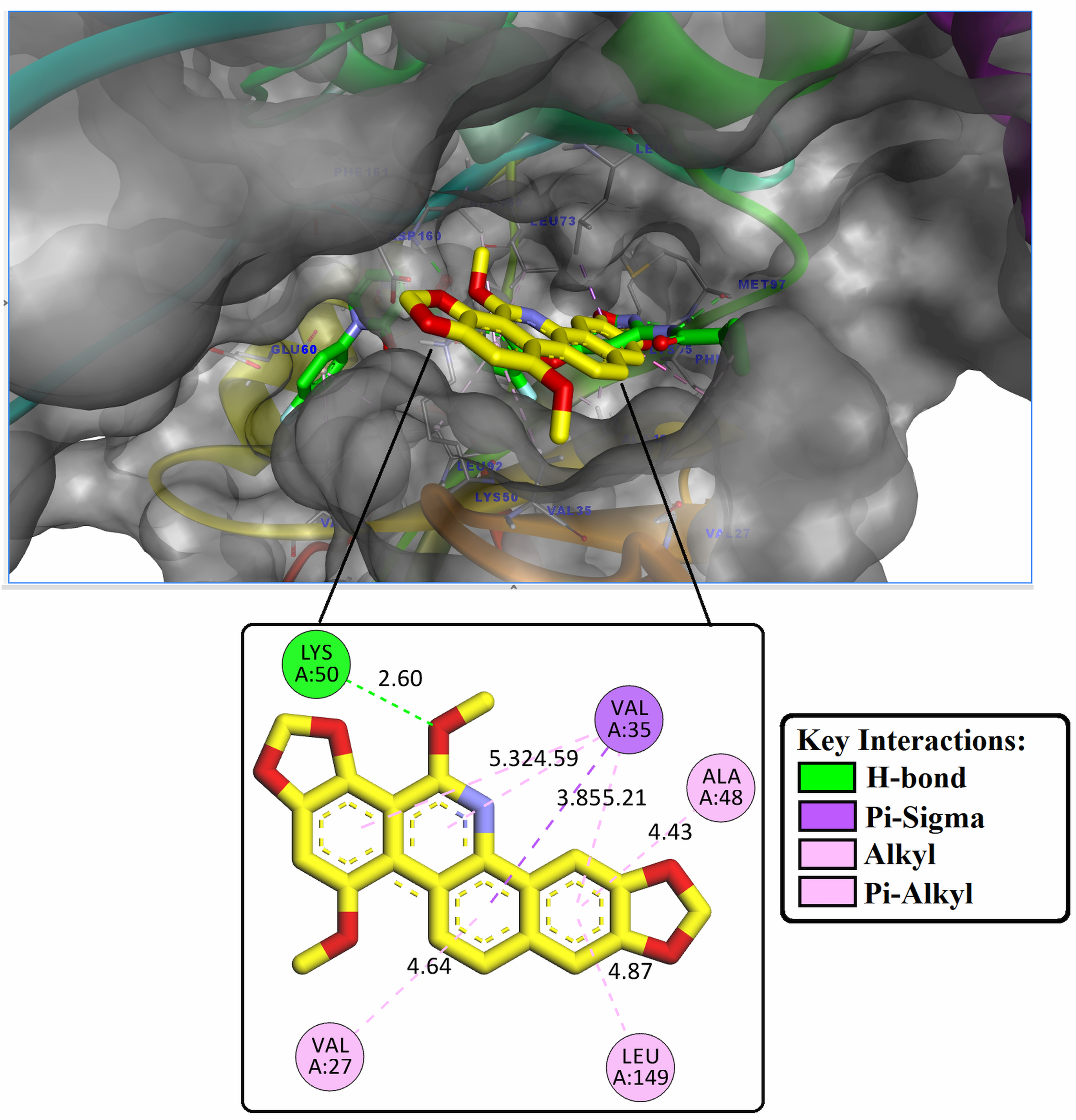

Supplement: S5 Fig — The co-crystallized ligand is shown with carbon atoms in green, oxygen in red, and nitrogen in blue, while SG-A is represented with carbon atoms in yellow, oxygen in red, and nitrogen in blue. (TIF) [file pone.0313094.s007.tif]
